# Multidimensional MRI Reveals Cellular‐Scale Microstructural Phenotypes in Human Brain Aging

**DOI:** 10.1111/acel.70782

**Published:** 2026-11-03

**Authors:** Joon Sik Park, Eppu Manninen, Shunxing Bao, Bennett A. Landman, Yihong Yang, Dan Benjamini

**Affiliations:** ^1^ Multiscale Imaging and Integrative Biophysics Unit, National Institute on Aging NIH Baltimore Maryland USA; ^2^ Department of Electrical and Computer Engineering Vanderbilt University Nashville Tennessee USA; ^3^ Neuroimaging Research Branch, National Institute on Drug Abuse NIH Baltimore Maryland USA

**Keywords:** aging, diffusion, magnetic resonance imaging, microstructure, multidimensional MRI, relaxation

## Abstract

Brain aging is accompanied by profound cellular and microstructural changes that precede overt tissue loss, yet in vivo MRI studies largely emphasize macroscopic measures or isolated diffusion and relaxation metrics, providing limited insight into how cellular‐scale tissue architecture is altered across the adult lifespan. Here, we apply multidimensional diffusion–relaxation MRI (MD‐MRI) to map voxel‐wise microstructural phenotypes in cognitively unimpaired adults spanning early adulthood to late life (23–77 years). Rather than relying on predefined compartment models, MD‐MRI resolves continuous voxel‐wise distributions in a joint diffusion–relaxation space, enabling an integrated, model‐free description of how cellular shape, size, restriction, and chemical environment vary with age. Using this approach, we reveal age‐related multilateral shifts within a complex microstructural landscape, marked by increasing heterogeneity and disorder across brain tissue. With age, cellular‐scale features showed a systematic transition from small to larger length‐scale structures accompanied by reduced microscopic restriction, indicating a loss of fine cellular barriers and expansion of extracellular space. In parallel, we show tissue‐dependent alterations in fast‐relaxing properties aligned with known gray‐ and white‐matter aging processes, including iron accumulation and myelin loss. Together, these findings indicate that normative brain aging involves progressive reorganization of structure and composition at the cellular level, rather than uniform shifts in bulk tissue properties. By decoupling cellular‐scale shape, size, restriction, and chemical environment in vivo, MD‐MRI identifies increasing cellular heterogeneity and breakdown of microscopic restriction as central features of human brain aging and provides a biologically interpretable framework for linking microstructural reorganization to age‐related functional decline.

## Graphical abstract

Multidimensional diffusion–relaxation MRI reveals that normative brain aging involves progressive reorganization of cellular‐scale microstructure—marked by expanding length‐scales, increasing heterogeneity, and breakdown of microscopic restriction—across cortical and subcortical gray matter and white matter throughout the adult lifespan.

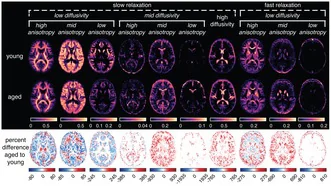

## 1 Introduction

Age‐related alterations in brain structure occur across multiple spatial scales and influence cognitive and functional trajectories throughout adulthood (Raz and Rodrigue 2006). At the macroscopic level, structural MRI studies consistently demonstrate cortical thinning and progressive volume loss in both superficial and deep gray matter (GM), with many of these changes accelerating during midlife and continuing into later decades (Resnick et al. 2003; Storsve et al. 2014). White matter (WM) undergoes parallel degeneration, including reduced tract orientational coherence, decreased myelination (Qian et al. 2020), and disruptions across projection, association, and commissural pathways (Schilling et al. 2022). Together, these findings highlight widespread structural remodeling of the aging brain, but largely reflect late‐stage or cumulative changes.

At the microscopic level, postmortem histological studies reveal a richer and more complex picture of brain aging. Hallmark features include dendritic regression, synaptic pruning, glial remodeling, and gradual expansion of the extracellular space, reflecting coordinated yet heterogeneous alterations in cytoarchitecture and tissue microstructure (Dickstein et al. 2013; Popov et al. 2021). These cellular‐level processes are thought to emerge early and to contribute to age‐related changes in neural function, long before overt tissue loss becomes apparent.

Diffusion MRI (dMRI) provides one of the primary noninvasive tools for interrogating brain microstructure by measuring water motion constrained by cellular and extracellular boundaries (Callaghan 2011). Conventional models such as diffusion tensor imaging (DTI) (Basser et al. 1994) robustly demonstrate age‐related increases in mean diffusivity and decreases in fractional anisotropy in white matter (Schilling et al. 2022). More recent biophysical models, including NODDI (Zhang et al. 2012) and SANDI (Palombo et al. 2020), aim to increase biological specificity and suggest age‐related reductions in the neurite density model parameter (Lee et al. 2024), often interpreted as reflecting demyelination, neurite loss, or extracellular space expansion. However, these models rely on strong a priori assumptions about tissue composition and geometry, limiting their validity in heterogeneous or weakly anisotropic regions such as cortical gray matter (Afzali et al. 2021; Jelescu et al. 2020). As brain aging involves multiple concurrent and spatially overlapping microstructural processes, collapsing this complexity into a small set of summary parameters may obscure subtle or early changes critical for understanding normative aging.

Growing evidence suggests that microstructural degeneration precedes measurable reductions in cortical or subcortical volume, even in cognitively unimpaired individuals (Rathi et al. 2014; Singh et al. 2024). This temporal dissociation underscores the need for noninvasive imaging approaches capable of detecting subtle and spatially heterogeneous microstructural changes before overt anatomical decline occurs. Approaches that resolve intra‐voxel heterogeneity are particularly valuable, as brain tissue contains diverse water environments that vary in cellular density, membrane properties, extracellular content, diffusion behavior, and relaxation characteristics (Qian et al. 2020; Syková and Nicholson 2008). Such heterogeneity cannot be adequately captured by unidimensional imaging measures but is essential for characterizing latent tissue remodeling during normal aging. While relaxometry studies have documented age‐related changes in T_1_, T_2_, and iron content across brain regions (MacDonald and Pike 2021), and b‐tensor encoding has shown that microscopic anisotropy decreases with age in white matter of older adults (Kamiya et al. 2020; Olsson et al. 2025), these two lines of evidence have been pursued independently and in isolation from one another.

Recent technological and theoretical advancements have led to an imaging framework that can address these limitations by merging two important aspects: simultaneous sensitivity to multiple MR contrasts and the ability to resolve their distribution within tissue. This strategy, known as multidimensional MRI (MD‐MRI), departs from conventional diffusion or relaxation imaging, which assigns each voxel a single averaged value, by acquiring data linked to multiple properties—typically diffusion tensors together with longitudinal (*R*_1_) and transverse (*R*_2_) relaxation rates—and reconstructing how these parameters are distributed rather than collapsing them into a mean value (Benjamini and Basser 2020; Slator, Palombo, et al. 2021). In practice, MD‐MRI can separate contributions from water pools with distinct microstructural phenotypes without enforcing specific compartment models, reducing partial‐volume effects and isolating environments that scalar maps blend together (English et al. 1991). Hardware and sequence innovations have made these multidimensional experiments feasible in vivo and ex vivo (Benjamini and Basser 2016; De Almeida Martins and Topgaard 2018; Kim et al. 2017; Kundu et al. 2023), enabling applications that range from characterizing microstructural variation in healthy neural tissue (Benjamini and Basser 2017; De Almeida Martins et al. 2021) to detecting changes associated with inflammation, degeneration, and tumor biology (Benjamini et al. 2020; Naranjo et al. 2021; Slator et al. 2023). Notably, ex vivo MD‐MRI has uncovered glial‐related alterations in Alzheimer's disease that are not detectable with conventional diffusion or relaxation imaging (Barsoum et al. 2025).

The concept of tensor‐valued diffusion encoding was first demonstrated using multidirectional diffusion encoding approaches (Mori and Van Zijl 1995; Wong et al. 1995), subsequently generalized into a tensor‐valued diffusion encoding framework (Eriksson et al. 2015; Westin et al. 2016), and later combined with relaxometry in the human brain (Lampinen et al. 2020). Building on the MD‐MRI framework that combines *R*_1_ and *R*_2_ relaxometry with tensor‐valued diffusion encoding (Martin et al. 2021), a newly developed and efficient in vivo MD‐MRI protocol has recently been introduced (Johnson et al. 2024; Manninen et al. 2024). This approach enables measurement of the frequency‐dependent diffusion spectrum, ***D*** (ω), which probes time‐dependent diffusion restrictions shaped by cellular morphology (Narvaez et al. 2022). In the current study, we leverage this novel MRI framework to chart the microstructural landscape of the aging brain across the adult lifespan. Using data from 46 cognitively unimpaired adults aged 23–77 years, we derive microstructural empirical “phenotype” based on cellular‐level shape (microscopic anisotropy), size (isotropic diffusivity), and chemical environment (*R*_2_ relaxation), along with restriction (diffusion frequency dependence). Because these phenotypes do not depend on biophysical modeling, they can be quantified across any brain region, as demonstrated here in cortical GM, deep GM nuclei, and major WM pathways. Our central hypothesis is that aging drives a systematic and diverse redistribution of diffusion‐relaxation subdomains. We find age‐related changes across multiple tissue characteristics: shifts from small toward both intermediate and large length‐scale phenotypes in GM, high‐to‐medium anisotropy phenotypes shift in WM, and low‐to‐high *R*_2_ relaxation phenotypes shift in both cortex and subcortex. Together, this framework enables a unified, cellular‐scale description of brain aging by framing age‐related changes as a coordinated redistribution of microstructural features, thereby linking in vivo MRI measurements to cellular processes known to underlie normative brain aging.

## 2 Methods

### 2.1 Participants

A total of 46 cognitively unimpaired healthy participants (20–77 years; mean age ± SD = 47.3 ± 17.6 years; 23 females) were drawn from ongoing control cohorts at the National Institute on Drug Abuse (NIDA). Participants were stratified into three age groups for descriptive and visualization purposes: young (20–31 years), middle‐aged (32–57 years), and aged adults (61–77 years), as detailed in Table 1. All individuals provided informed consent prior to participation in the study, which was approved by the NIDA Institutional Review Board. Exclusion criteria included major medical illness or current medication use, a history of neurological or psychiatric disorders, or any history of substance abuse.

**TABLE 1:** Demographic characteristics of the participants.

Group	Sample size	Sex (F/M)	Mean age ± SD (years)	Age range (years)
Young	17	9/8	25.6 ± 3.4	20–31
Middle	15	7/8	45.1 ± 6.2	32–57
Aged	14	7/7	67.3 ± 5.9	61–77
Total	46	23/23	47.3 ± 17.6	20–77

### 2.2 Data Acquisition

MRI data were acquired on a 3 T MAGNETOM Prisma scanner (Siemens Healthineers, Erlangen, Germany) using a 32‐channel receive array. Multidimensional diffusion‐relaxometry measurements were obtained with a single‐shot spin‐echo EPI sequence incorporating tensor‐valued diffusion encoding generated by numerically optimized free gradient waveforms. Images were acquired at 2 mm isotropic resolution (FOV = 228 × 228 × 110 mm^3^, bandwidth = 1512 Hz/pixel), with GRAPPA acceleration (factor = 2, 24 reference lines), an effective echo spacing of 0.8 ms, partial Fourier 6/8, and axial slice orientation (Johnson et al. 2024). The limited field of view in the inferior–superior direction prevents whole‐brain analyzes.

The diffusion‐encoding scheme followed a sparse yet information‐rich sampling strategy. In addition to a non‐diffusion‐weighted (*b* = 0) volume, the protocol included linear ($b_{∆}$= 1), spherical ($b_{∆}$ = 0), and planar ($b_{∆}$ = −0.5) b‐tensors spanning b‐values from 0.1 to 3 ms/μm^2^. Each waveform was characterized by a well‐defined diffusion spectral profile, with centroid frequencies ($\omega_{\text{cent}}$/2π) ranging from 6.6 to 21 Hz, enabling sensitivity to time‐dependent diffusion across biologically relevant frequency bands (Yon et al. 2025). The diffusion encoding gradient waveforms used in this study were asymmetric with respect to the refocusing pulse, which introduces the potential for concomitant gradient fields to cause spatially varying signal bias (Baron et al. 2012; Szczepankiewicz et al. 2019). This potential bias was characterized for our specific waveforms and acquisition parameters; the analysis is provided in Supporting Information S1.

Relaxation sensitivity was achieved by varying repetition times (TR = 0.62, 1.75, 3.5, 5, 7, and 7.6 s) and echo times (TE = 40, 63, 83, and 150 ms), resulting in 139 unique diffusion‐relaxation encoding combinations. The total MD‐MRI acquisition time was approximately 40 min. Images were acquired with an anterior‐to‐posterior phase‐encoding direction, and an additional reversed (*b* = 0) posterior‐to‐anterior volume was collected to enable susceptibility‐induced distortion correction. Further details are provided in the Supporting Information S1 and in Figure S1.

High‐resolution structural reference images were acquired using a fat‐suppressed $T_{1}$‐weighted MPRAGE sequence (TR = 1900 ms, TE = 3.42 ms, 1 mm isotropic resolution). These anatomical images were used for co‐registration, brain extraction, and region‐of‐interest definition.

### 2.3 Data Preprocessing

MD‐MRI data were preprocessed following current recommendations for sparse MD‐MRI imaging (Johnson et al. 2024). All diffusion‐weighted volumes were first concatenated and denoised using the Marchenko‐Pastur principal component analysis (MP‐PCA) method (Veraart et al. 2016). Motion and eddy‐current distortions were corrected using the DIFFPREP module of the TORTOISE software package (Irfanoglu et al. 2023), employing a physically based quadratic transformation model and a normalized mutual information similarity metric. Susceptibility‐induced geometric distortions were corrected with the DR‐BUDDI algorithm (Irfanoglu et al. 2015) by providing the acquired anterior–posterior (AP) and posterior–anterior (PA) phase‐encoding *b* = 0 images together with a synthetic $T_{2}$‐weighted (*b* = 0‐like) image generated from the $T_{1}$‐weighted anatomical scan (Schilling et al. 2019). All datasets underwent visual quality‐control assessment following preprocessing. Quantitative head motion was estimated from the rigid‐body motion parameters generated by TORTOISE and summarized as the mean relative displacement between consecutive diffusion volumes. Motion levels were low across the cohort (0.24–0.85 mm), and additional details are provided in the Supporting Information S1.

### 2.4 Multidimensional Data Processing

Preprocessed MD‐MRI data were analyzed in MATLAB (MathWorks, Natick, MA) using a Monte Carlo inversion framework implemented in the frequency dependent multidimensional diffusion MRI toolbox (Narvaez et al. 2022). The measured signal was modeled as a weighted superposition of discrete components spanning the joint diffusion‐relaxation parameter space:

(1) $$S\left(b\left(\omega \right),\mathrm{TE},\mathrm{TR}\right)=\sum_{c=1}^{N_{c}}f_{i}\exp\left(-\int_{-\infty }^{\infty }b\left(\omega \right):D_{i}\left(\omega \right)\;d\omega \right)\left(1-e^{-\mathrm{TR}\cdot R_{1,i}}\right)e^{-\mathrm{TE}\cdot R_{2,i}}$$

where $f_{i}$ denotes the weight of the i‐th component and: represents the generalized scalar product. Each frequency‐dependent diffusion tensor $D_{i}\left(\omega \right)$ was modeled as an axisymmetric Lorentzian function parameterized by axial diffusivity $D_{∥,i}$, radial diffusivity $D_{⊥,i}$, high‐frequency isotropic diffusivity $D_{0,i}$, orientation angles ($\theta_{i}$, $\phi_{i}$), axial and radial transition frequencies ($\Gamma_{∥,i}$ $\Gamma_{⊥,i}$), and longitudinal and transverse relaxation rates ($R_{1,i}$, $R_{2,i}$) (De Almeida Martins and Topgaard 2018; Narvaez et al. 2022; Johnson et al. 2024). For convenience in interpretation, axial and radial diffusivities are expressed here as isotropic diffusivity, $D_{\mathrm{iso}}$, = ($D_{∥}$ + $2D_{⊥}$)/3, and squared microscopic diffusion anisotropy, $D_{∆}^{2}=\frac{{\left(D_{∥}-D_{⊥}\right)}^{2}}{{\left(D_{∥}+2D_{⊥}\right)}^{2}}$. We computed voxel‐wise means and variances for all diffusion‐relaxation parameters, denoted as E[·] and V[·] (e.g., E[$D_{\mathrm{iso}}$]), respectively. Following conventions, diffusion frequency dependence was quantified by finite‐difference slopes computed across the sampled centroid frequency range (i.e., between $\omega_{\min}$ = 6.6 Hz and $\omega_{\max}$ = 21 Hz), noted as ${∆}_{\omega /2\pi }$ E[$D_{\mathrm{iso}}$] and ${∆}_{\omega /2\pi }$ E[$D_{∆}^{2}$]for isotropic diffusivity and anisotropy, respectively (Johnson et al. 2024; Narvaez et al. 2022). Full technical details regarding implementation are provided in the Supporting Information S1.

### 2.5 Definition of Diffusion‐Relaxation Subdomains

By treating $D\text{(}\omega \text{)}‐R_{1}‐R_{2}$ distributions as spectra, images of individual spectral components can be obtained through integration over predefined diffusion–relaxation parameter subdomains. The resulting value, ranging from 0 to 1, represents the signal fraction of that component and can be computed voxel‐wise to produce spatial maps of specific microstructural “phenotypes”, based on their length‐scale, shape, and chemical microenvironment. This approach has previously been used to probe microscopic eccentricity (Benjamini and Basser 2014), diffusivity–*T*_2_ subdomains (Benjamini et al. 2023; Benjamini and Basser 2017; Pas et al. 2020; Slator, Hutter, et al. 2021) and diffusivity–anisotropy subdomains in vivo (De Almeida Martins et al. 2020; Manninen et al. 2024; Martin et al. 2021). Here, we combine these strategies by partitioning the continuous dimensions of $D_{\mathrm{iso}}$, $D_{∆}^{2}$ and $R_{2}$ into subdomains, based on heuristic principles:

- The isotropic diffusivity in MD‐MRI is commonly partitioned into two subdomains (De Almeida Martins et al. 2020). Here, we expand that division into three subdomains: low (0.05 < $D_{\mathrm{iso}}$< 1.5 μm^2^/ms), intermediate (1.5 < $D_{\mathrm{iso}}$ < 3 μm^2^/ms), and high ($D_{\mathrm{iso}}$ > 3 μm^2^/ms) spectral subdomains, which capture small, intermediate, and large microscopic length‐scales, respectively.
- The diffusion microscopic anisotropy in MD‐MRI is commonly partitioned into two subdomains (De Almeida Martins et al. 2020). Here, we expand that division into three subdomains: low (0 < $D_{∆}^{2}$< 0.01), intermediate (0.01 < $D_{∆}^{2}$< 0.25), and high ($D_{∆}^{2}$ > 0.25) spectral subdomains, which capture low, intermediate, and high microscopic anisotropy, respectively.
- The $R_{2}$ relaxation is normally not partitioned in MD‐MRI. Here, we partition it to include low (1 < $R_{2}$ < 20 s^−1^) and high ($R_{2}$ > 20 s^−1^) spectral subdomains, which capture contributions from tissue water and macromolecule‐bound water, respectively (Alonso‐Ortiz et al. 2015).

In total, we define and investigate 10 $D_{\mathrm{iso}}-D_{∆}^{2}-R_{2}$ subdomains, which we term here “microstructural phenotypes”. For convenience, we use the left superscripts l, m, and h to denote low, intermediate, and high ranges along each parameter dimension, respectively. For example, the phenotype |
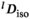
|
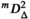
|
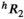
| represents the normalized signal fraction of a microstructural phenotype with low isotropic diffusivity, intermediate diffusion anisotropy, and high $R_{2}$ relaxation. For the 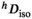 range, we include the full anisotropy range as previously established (De Almeida Martins et al. 2020; Manninen et al. 2024; Martin et al. 2021), and denote it as |
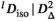
|
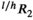
|. Further details are provided in the Supporting Information S1.

These microstructural phenotypes are diffusion‐frequency dependent. However, throughout most of this work we analyze them at the lowest diffusion frequency ($\omega_{\min}$ = 6.6 Hz) and therefore omit the $\omega_{\min}$ notation for clarity. When explicitly examining frequency dependence, we use $\omega_{\min}$ and $\omega_{\max}$ to indicate the frequencies at which the phenotypes were derived.

Because these subdomain boundaries were chosen heuristically, we assessed the robustness of our age associations to the specific threshold values by repeating the full analysis across 243 configurations in which each threshold was independently shifted by ±10% (see Supporting Information S1 for full analysis).

### 2.6 Noise Floor Considerations

Signal‐to‐noise ratio (SNR) was estimated per subject, voxel, and measurement to assess potential noise floor effects. The noise standard deviation σ was derived per subject from a background region of interest (ROI) containing no tissue signal. In the absence of true signal, magnitude MRI data follow a Rayleigh distribution, for which the standard deviation equals σ√(2 − π/2). The per‐measurement σ was therefore obtained by dividing the standard deviation of the background signal across voxels by √(2 − π/2) ≈0.655 (Gudbjartsson and Patz 1995), and averaged across all 139 measurements to yield a single *σ* per subject. SNR was then computed per individual voxel as the ratio of the voxel signal to *σ* at each measurement. To summarize across the ROI while preserving the per‐voxel SNR estimates, the median SNR across voxels within each ROI was reported. This differs from ROI SNR, which would be computed from the signal averaged across voxels and would overestimate the SNR experienced during voxel‐wise model fitting. The median SNR across subjects, taken at the most attenuated measurement and summarized per ROI, ranged from 8.7 to 27.0 across the 28 ROIs, well above the threshold of SNR = 2 below which Rician noise introduces meaningful signal bias (Gudbjartsson and Patz 1995). The full analysis is provided in the Supporting Information S1.

To further validate the robustness of the inversion pipeline, simulations were performed by adding Rician noise at the SNR levels observed in the study to synthetic signals generated from the forward model (Equation 1) with a canonical ground truth spectrum representative of brain tissue. Across 13 output parameters and 900 noise realizations, median relative bias was below 15% for 11 of 13 parameters; the two exceptions were diffusion anisotropy parameters (V[$D_{∆}^{2}$] and ${∆}_{\omega /2\pi }$ E[$D_{∆}^{2}$]), whose elevated bias is consistent with known gradient hardware limitations of clinical MRI scanners and does not reflect noise floor effects (see detailed simulations results in the Supporting Information S1, Figures S4 and S5). Nevertheless, noise floor could affect some of the observations, particularly in low SNR regions such as the putamen.

### 2.7 Image Registration and Whole Brain Segmentation

Cortical and subcortical GM regions were obtained using the spatially localized atlas network tiles (SLANT) method (Huo et al. 2019), which applies region‐specific 3D convolutional networks following standard preprocessing. The resulting segmentations yield 132 anatomical labels based on the BrainCOLOR atlas and have demonstrated high reproducibility. GM segmentation was performed on 1 mm $T_{1}$‐weighted images in native space and downsampled to 2 mm resolution to match the MD‐MRI data.

White matter tract labels were derived from the Johns Hopkins University (JHU) DTI‐based WM atlas using Advanced Normalization Tools (ANTs v2.5.2). Because direct registration from individual participant images to atlas space was inaccurate, age‐specific group templates were used as an intermediate step. Participants were assigned to one of three age groups (Table 1), and for each group a template was generated by co‐registering $T_{1}$‐weighted images using affine and symmetric normalization (SyN). Templates were down‐sampled to 2 mm isotropic resolution to match the MD‐MRI images and the JHU atlas fractional anisotropy map. Group‐averaged E[$D_{∆}^{2}$] images were registered to the JHU atlas using affine and SyN transforms. The resulting transforms—from participant to group template and from group template to atlas space—were concatenated, inverted, and applied to map atlas labels into each participant's native space, with a single interpolation used to minimize interpolation errors. The log Jacobian determinant of the group average‐to‐atlas deformation fields was comparable across all three age groups (young: 0.044 ± 0.345, middle: 0.054 ± 0.307, aged: 0.023 ± 0.315), with no evidence of systematically larger or more irregular deformations in the aged group, arguing against differential registration‐induced mislocalization of WM tract labels across age groups.

To enable voxel‐wise comparisons, MD‐MRI parameter maps were transformed to FSL's MNI152 template space. Group‐registered $T_{1}$‐weighted images (1 mm isotropic) were first registered to the MNI152 $T_{1}$‐weighted template (1 mm isotropic) using affine and SyN transforms. The resulting transforms—from participant space to group template and from group template to MNI152 space—were concatenated and applied to the MD‐MRI maps, with a single interpolation used to minimize interpolation errors.

In this study we chose to focus on a wide array of cortical regions of interest (ROI) that included the frontal pole (FRP), anterior orbital gyrus (AOrG), posterior orbital gyrus (POrG), opercular part of the inferior frontal gyrus (OpIFG), triangular part of the inferior frontal gyrus (TrIFG), middle frontal gyrus (MFG), medial precentral gyrus (MPrG), supplementary motor cortex (SMC), anterior insula (AI), superior temporal gyrus (STG), postcentral gyrus (PoG), posterior cingulate gyrus (PCgG), precuneus (PCu), angular gyrus (AnG), middle occipital gyrus (MOG), cuneus, and entorhinal cortex (EntC). Subcortical ROIs included the hippocampus, caudate, putamen, and thalamus. White matter tracts included the genu of the corpus callosum (genu CC), anterior corona radiata (ACR), superior corona radiata (SCR), sagittal stratum (SS), external capsule (EC), cingulum–hippocampus bundle (CH), and the superior fronto‐occipital fasciculus (SFOF). Left and right ROIs were combined to create single regions. In total, 28 ROIs were investigated.

### 2.8 Statistical Analysis

Statistical analyzes were performed in MATLAB (MathWorks, Natick, MA) to assess age‐related effects on MD‐MRI microstructural phenotypes across cortical, subcortical, and WM ROIs. Age associations were evaluated using a quadratic regression model applied independently to each MD‐MRI parameter and ROI. Sex was included as a covariate using effect coding (male = 0.5, female = −0.5). The regression model was defined as $P_{r}=\beta_{0}+\beta_{\mathrm{sex}}\times \mathrm{sex}+\beta_{\mathrm{age}}\times \mathrm{age}+\beta_{{\mathrm{age}}^{2}}\times {\mathrm{age}}^{2}$, where $P_{r}$ denotes the ROI‐averaged MD‐MRI parameter of interest (e.g., E[$D_{\mathrm{iso}}$], |
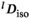
|
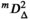
|
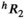
|, etc.) for region r. Linear age effects were quantified by $\beta_{\mathrm{age}}$, while nonlinear lifespan trajectories were assessed via the quadratic term $\beta_{{\mathrm{age}}^{2}}$. All continuous variables were z‐standardized prior to regression, such that regression coefficients represent standardized effect sizes.

Multiple‐comparison correction across all ROIs and MD‐MRI metrics was performed using the Benjamini–Hochberg false discovery rate (FDR) procedure, with statistical significance defined as *p*FDR < 0.05 (Benjamini and Yekutieli 2001).

## 3 Results

### 3.1 Interpretation Framework for Microstructural Phenotypes

Unlike biophysical compartment models such as NODDI or SANDI—which link each parameter to predefined geometries (e.g., spheres for soma, sticks for neurites) and a fixed number of intra‐voxel compartments—MD‐MRI estimates the distribution of diffusion‐relaxation components directly from the measured signal, without assuming any specific tissue geometry or compartment count. Consequently, the derived parameters should be interpreted as phenomenological descriptors of diffusion and relaxation behavior, rather than as direct quantitative measures of specific cellular constituents.

While MD‐MRI provides voxel‐wise joint distributions of frequency‐dependent diffusion tensors D(ω) and relaxation parameters $R_{1}$ and $R_{2}$, we choose here to focus on a projection onto the 3D parameter space spanned by the isotropic diffusivity, $D_{\mathrm{iso}}$, the squared microscopic diffusion anisotropy, $D_{∆}^{2}$, and the transverse relaxation rate $R_{2}$. Together, these three parameters define a multidimensional diffusion‐relaxation space in which distinct signal components occupy characteristic subdomains. Hence, we define the term “microstructural phenotype” to describe the normalized signal fraction from predefined ranges within the $D_{\mathrm{iso}}$–$D_{∆}^{2}-R_{2}$ distribution. As detailed in the Methods Section, we partitioned the continuous dimensions of $D_{\mathrm{iso}}$, $D_{∆}^{2}$, and $R_{2}$ into three, three, and two subdomains, based on heuristic principles. Here, the left superscripts l,m, and h denote low, intermediate, and high ranges along each parameter dimension, respectively, as illustrated in Figure 1A. For example, the phenotype |
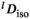
|
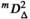
|
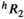
| represents the normalized signal fraction of a microstructural phenotype with low isotropic diffusivity, intermediate diffusion anisotropy, and high $R_{2}$ relaxation.

**FIGURE 1:**
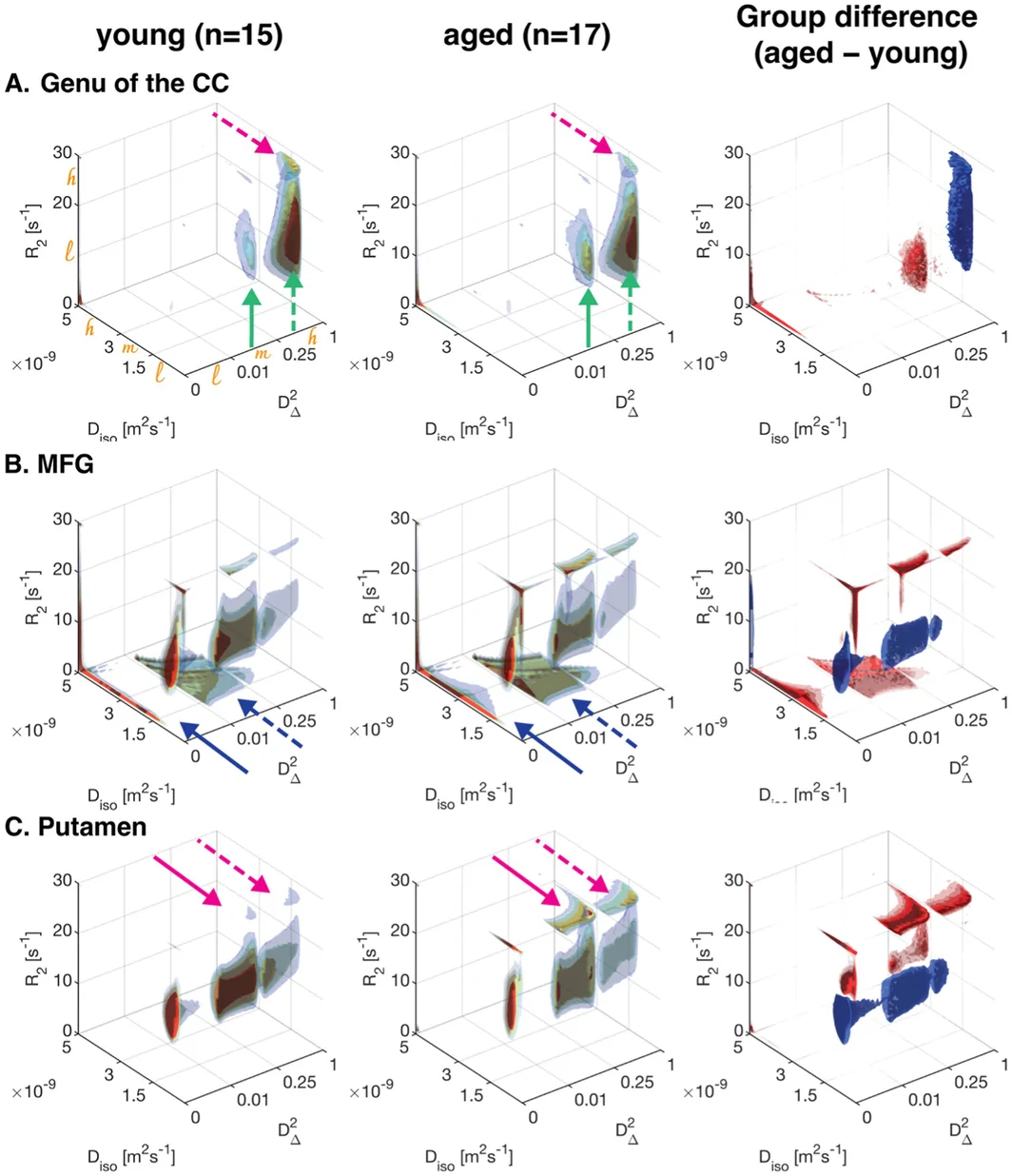
Age‐related redistribution of joint diffusion‐relaxation phenotypes across major brain tissue classes. Group‐averaged three‐dimensional joint $D_{\mathrm{iso}}-D_{∆}^{2}-R_{2,}$ distributions projected at minimal frequency, $\omega_{\min}$ = 6.6 Hz, are shown for young (left column), and aged adults (middle column) in the (A) genu of the corpus callosum, (B) middle frontal gyrus (MFG), and (C) putamen. Nested isosurfaces are shown at the 98th, 99th, 99.5th, and 99.9th percentiles of the grand‐mean distribution pooled across all subjects, colored from deep blue (lowest) to red (highest) with opacity increasing cubically with percentile level. On the right column, red (positive) and blue (negative) isosurfaces show regions of difference between aged and young adults, rendered at four opacity levels spanning 50–100% of the peak absolute difference. White surface indicates zero. Across brain regions, aging is marked by multilateral microstructural shifts that reflect complex trajectories and together form a shared aging signature. Low, intermediate, and high phenotype ranges, l, m, and h, are indicated in (A). Arrows indicate major age‐related shifts in microstructural phenotypes.

Importantly, because these microstructural phenotypes sum to 1 within each voxel, any age‐related effects can be assessed and interpreted as systematic redistributions across phenotypes within the joint $D_{\mathrm{iso}}-D_{∆}^{2}-R_{2}$ space, rather than as changes in predefined biological compartments.

### 3.2 Multilateral Diffusion–Relaxation Shifts Reveal Complex Age‐Related Microstructural Change

The participants were grouped into three categories reflecting different phases of adult life: young adults (*n* = 15), middle‐aged adults (*n* = 14), and aged adults (*n* = 17). Figure 1 illustrates group differences and age effects in microscopic length scale, shape, and chemical composition reflected in the joint $D_{\mathrm{iso}}-D_{∆}^{2}-R_{2}$ distributions projected on the lowest frequency $\omega_{\min}$ = 6.6 Hz, for representative brain regions spanning major tissue classes, including a commissural WM tract (genu of the corpus callosum), cortical GM (middle frontal gyrus), and subcortical GM (putamen), shown in Figure 1A–C, respectively. For each region, the right column shows the aged‐minus‐young difference map, where red and blue isosurfaces indicate phenotypes more prominent in aged and young adults, respectively. These regions were selected to illustrate how age‐related microstructural alterations manifest across tissues with distinct organizational architectures, ranging from highly anisotropic fiber bundles to heterogeneous cortical and subcortical regions.

In the genu of the corpus callosum (Figure 1A), younger adults exhibited a dominant diffusion‐relaxation phenotype characterized by low isotropic diffusivity, high microscopic diffusion anisotropy (dashed green arrow), and a fast $R_{2}$ component (dashed pink arrow), occupying a compact region of the joint space. With increasing age, this distribution systematically shifts toward the intermediate anisotropy $D_{∆}^{2}$ range (solid green arrow), and reduced intensity of the fast $R_{2}$ component (dashed pink arrow), indicating a progressive reduction in microstructural restriction and directional coherence. This is reflected in the difference map as a blue region at high $D_{∆}^{2}$ and a red region at intermediate $D_{∆}^{2}$, both at low $D_{\mathrm{iso}}$. Notably, this change was expressed as a shift of the dominant phenotype |
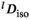
|
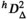
|
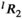
| toward |
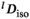
|
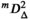
|
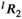
|, rather than the emergence of a qualitatively new diffusion‐relaxation component.

The middle frontal gyrus region (Figure 1B) showed broader, more heterogeneous distributions across all age groups, consistent with the inherently less ordered cortical microarchitecture. We observed a complex age‐related pattern across microstructural phenotypes: small length‐scale phenotypes (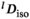) across all shapes shifted toward intermediate and large diffusivity ranges, mainly within low and intermediate anisotropy ranges (solid and dashed blue arrows, respectively). In addition, a buildup of the small length‐scale and fast $R_{2}$ phenotypes can be seen. The difference map confirms this complexity, showing interleaved red and blue regions reflecting competing redistributions across the joint space rather than a unidirectional shift.

In the putamen (Figure 1C), redistribution followed a distinct pattern, with prominent age‐related increases in the small length‐scale and fast $R_{2}$ phenotypes (dashed and solid pink arrows for high and intermediate anisotropy, respectively). A concomitant age‐related decrease in low $R_{2}$ phenotypes was observed, along with a general shift from higher to lower microstructural anisotropy. The difference map clearly captures this pattern, with red isosurfaces concentrated at high $R_{2}$ and blue isosurfaces at low $R_{2}.$ This shift was more pronounced along the relaxation dimension than in cortical GM, suggesting that age‐related changes in the macromolecular environment and water–tissue interactions may play a particularly important role in age‐related microstructural remodeling within deep GM nuclei.

The corresponding distributions evaluated at the highest diffusion frequency ($\omega_{\max}$ = 21 Hz) are shown in the Supporting Information S1 (Figure S6), illustrating how microscopic length scale, shape, and chemical composition vary with diffusion frequency across the same representative white‐ and gray‐matter regions. An explicit frequency dependence analysis is provided in Section 3.7.

### 3.3 Age Group Differences in Diffusion‐Relaxation Parameter Maps

Multidimensional MRI delivers voxel‐wise distributions like the ones seen in Figure 1. The information can be most straightforwardly summarized by mapping weighted averages and variances across the different dimensions. Such group‐averaged maps are shown in Figure 2A, along with corresponding aged‐to‐young percentage difference maps (Figure 2B), where *E*[·] and *V*[·] denote the voxel‐wise average and variance of a given dimension, respectively. In addition, ${∆}_{\omega /2\pi }$ *E*[·] captures the diffusion frequency dependence of the corresponding diffusion metrics.

**FIGURE 2:**
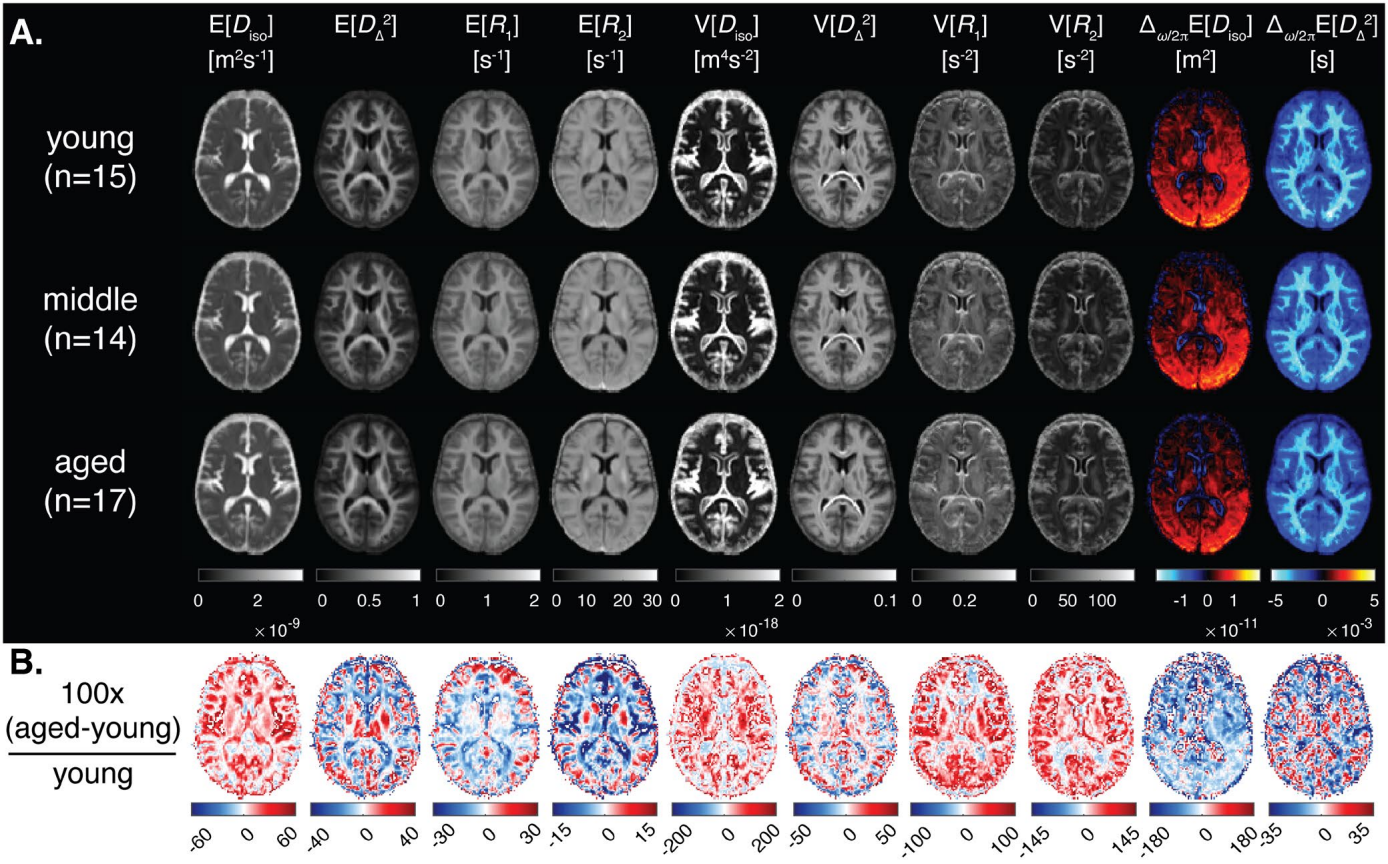
Diffusion–relaxation parameters shown in (A) as participant‐averaged maps for each age group. Panel (B) presents percent change maps for each metric, highlighting differences between the young (20–32 years) and aged (61–77 years) groups. Displayed parameters include expectation values *E*[$D_{\mathrm{iso}}$], *E*[$D_{∆}^{2}$], *E*[$R_{1}$] and *E*[$R_{2}$], corresponding to MD, microscopic anisotropy, 1/*T*_2_, and 1/*T*_2_; the corresponding variances *V*[$D_{\mathrm{iso}}$], *V*[$D_{∆}^{2}$], *V*[$R_{1}$], and *V*[$R_{2}$], which capture intra‐voxel parameter heterogeneity; and the diffusion‐frequency slopes ${∆}_{\omega /2\pi }$ *E*[$D_{\mathrm{iso}}$] and ${∆}_{\omega /2\pi }$ *E*[$D_{∆}^{2}$], which reflect microstructural restriction.

The average parameter maps *E*[$D_{\mathrm{iso}}$], E[$D_{∆}^{2}$], *E*[$R_{1}$] and *E*[$R_{2}$] roughly correspond to DTI's mean diffusivity, microscopic diffusion anisotropy, 1/*T*_1_, and 1/*T*_2_. Visual inspection shows that these metrics follow the expected age‐related trajectories, in which older adults exhibit higher mean isotropic diffusivity and lower microscopic anisotropy. Mean relaxometry parameters showed opposing age‐related trends in GM and WM, increasing with age in GM and decreasing in WM (Figure 2B).

Beyond differences in mean values, variance maps (i.e., *V*[·]) demonstrated pronounced age‐related increases in all parameters, particularly in frontal and parietal association cortices, reflecting heightened intra‐voxel heterogeneity and greater dispersion of microstructural environments in aging tissue. Complementing these findings, diffusion frequency slopes, ${∆}_{\omega /2\pi }$ *E*[$D_{\mathrm{iso}}$], and ${∆}_{\omega /2\pi }$ *E*[$D_{∆}^{2}$], exhibited systematic flattening with age, suggestive of attenuated restriction‐driven frequency dependence and increased extracellular contributions.

Region‐of‐interest–averaged MD‐MRI diffusion–relaxation parameters, stratified by age group, are given in Figure S7.

### 3.4 Age Group Differences in Microstructural Phenotype Maps

The most distinctive and powerful feature of MD‐MRI is its ability to resolve diffusion–relaxation subdomains within a voxel, yielding effective intra‐voxel microstructural resolution. Maps of these subdomains directly visualize how signal weight redistributes across distinct regions of the joint diffusion–relaxation space. Rather than representing changes in predefined model‐based biological compartments, they capture age‐related shifts in the relative contributions of different diffusion–relaxation subdomains. Importantly, these quantities sum to 1 as they represent the normalized signal fraction from all subdomains. These microstructural phenotypes are shown in Figure 3, which reveals pronounced age‐related redistribution of signal contributions within the joint diffusion–relaxation space. Because these novel microstructural phenotypes follow opposing age‐related trajectories in cortical and subcortical GM and WM regions, we next examine each of them separately.

**FIGURE 3:**
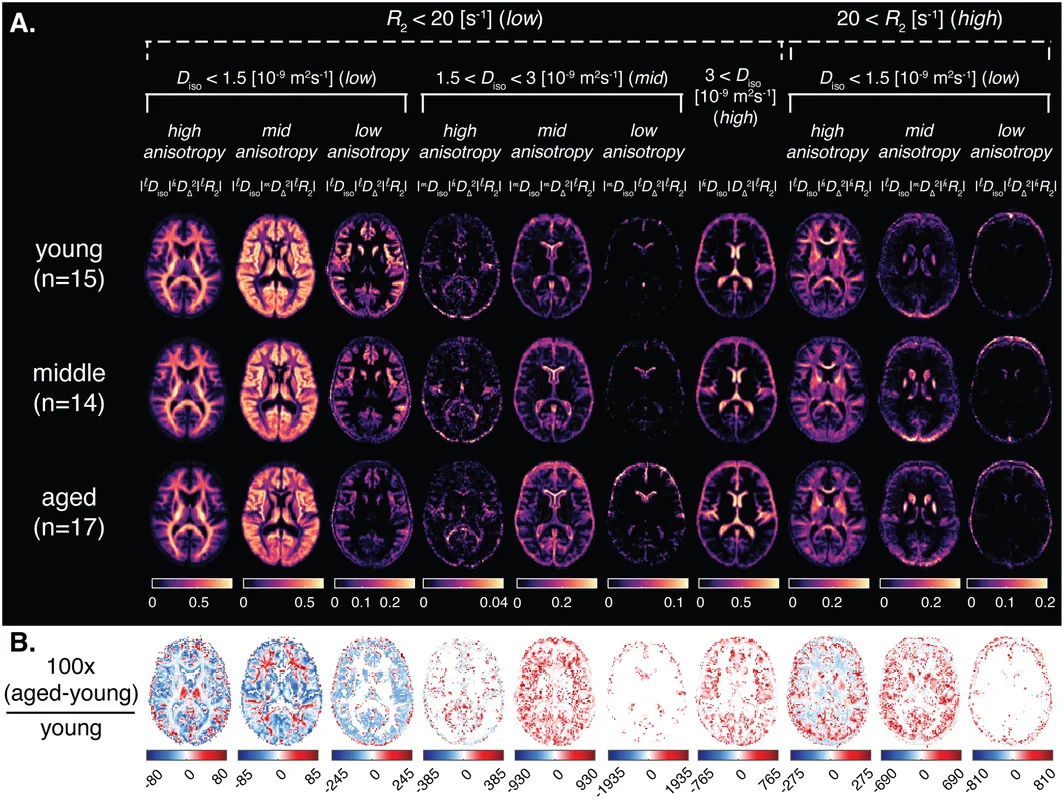
Microstructural phenotypes shown in (A) as participant‐averaged maps for each age group. Panel (B) presents percent change maps for each metric, highlighting differences between the young (20–32 years) and aged (61–77 years) groups. These phenotypes represent the normalized signal contributions of distinct subdomains of the joint $D_{\mathrm{iso}}-D_{∆}^{2}-R_{2}$ space, defined by combinations of low (l), intermediate (m), and high (h) values of isotropic diffusivity, diffusion anisotropy, and *R*_2_ relaxation rate, without assuming correspondence to specific biological compartments. Group‐difference maps in (B) highlight widespread age‐related redistribution toward larger cellular length‐scales, increased macromolecular and iron content in GM, and reduced microscopic anisotropy.

In cortical and subcortical GM regions, aging was accompanied by an increase in the characteristic cellular length‐scale, whereas microscopic anisotropy was preserved. This pattern is reflected by negative aged‐to‐young relative differences in |
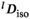
|
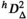
|
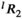
|, |
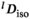
|
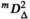
|
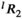
|, and |
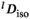
|
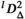
|
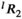
|, alongside concomitant positive aged‐to‐young relative differences in |
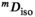
|
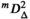
|
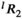
|, and |
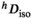
|$D_{∆}^{2}$|
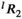
| (Figure 3B). Similarly, tissue phenotypes with fast $R_{2}$ relaxation and small cellular length‐scales show an increasing prevalence with age across the GM, indicative of increased macromolecular and iron content. The caudate and putamen show especially strong effects, with percent differences as high as 690%.

In WM, microstructural redistribution is more constrained, characterized by a decrease in highly anisotropic, small length‐scale tissue phenotypes and concurrent increases in intermediate‐anisotropy, small length‐scale and unrestricted, high‐diffusivity phenotypes. Specifically, |
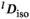
|
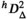
|
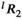
| shifts toward |
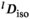
|
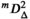
|
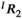
|, and |
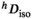
|$D_{∆}^{2}$|
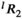
|. In addition, the prevalence of small length‐scale, high‐anisotropy, high‐R_2_ phenotypes is reduced, consistent with decreased macromolecular content (e.g., myelin).

Region‐of‐interest–averaged MD‐MRI microstructural phenotypes, stratified by age group, are given in Figure S8. Some phenotypes have clear regional specificity; for example, both |
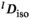
|
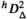
|
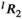
| and |
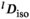
|
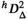
|
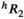
| are more prevalent in WM. Conversely, lower anisotropy subdomains, for example, |
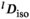
|
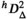
|
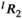
| or |
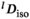
|
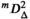
|
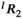
|, mainly dominate cortical and subcortical regions (Figure S8).

### 3.5 Linear and Nonlinear Age Associations in Diffusion‐Relaxation Metrics

To provide context for the phenotype‐level analysis that follows, and to establish consistency with the prior diffusion and relaxometry literature, we first examined both linear and quadratic associations with age across several diffusion–relaxation MD‐MRI parameters. Associations were expressed using z‐scored (standardized) coefficients, $\beta_{\mathrm{age}}$ and $\beta_{{\mathrm{age}}^{2}}$, which represent the change in the MR variable, in units of standard deviation, per year. The resulting age‐related effect sizes are shown in Figure 4A,B for the linear and quadratic associations, respectively. In addition, scatter plots from representative regions and MD‐MRI parameters are shown in Figure 4C.

**FIGURE 4:**
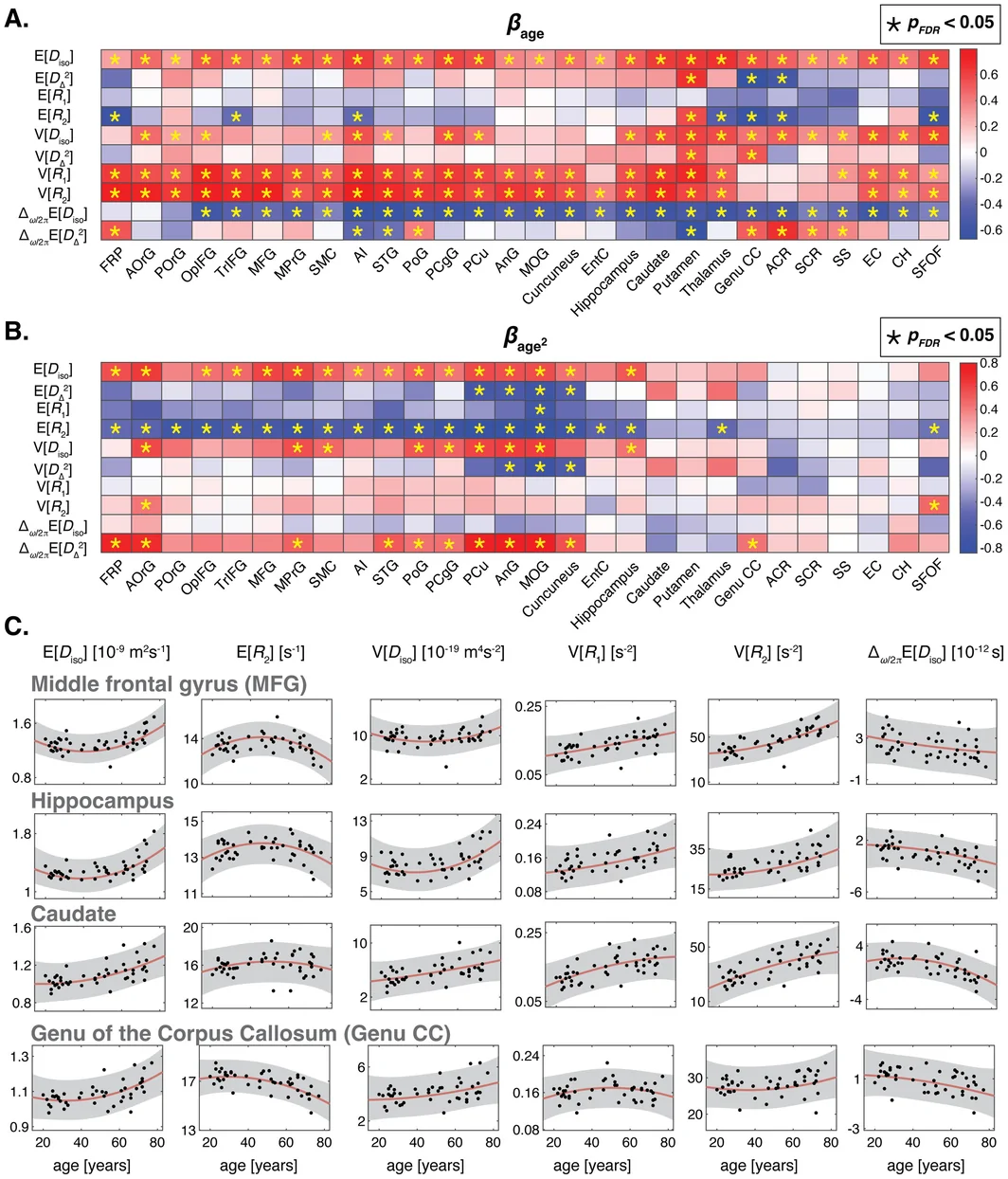
Linear and nonlinear age associations of multidimensional MD‐MRI parameters. (A) Standardized linear age coefficients ($\beta_{\mathrm{age}}$) for expectation values, variances, and diffusion‐frequency slopes of diffusion–relaxation parameters across cortical, deep GM, and WM ROIs. (B) Corresponding quadratic age coefficients ($\beta_{{\mathrm{age}}^{2}}$) capturing nonlinear lifespan effects. Asterisks indicate false‐discovery‐rate corrected significance (*p*_FDR_ < 0.05). Scatter plots from representative regions and MD‐MRI parameters are shown in panel (C). Solid lines denote model fits, with shaded regions indicating 95% confidence intervals.

Consistent with their distinct microstructural organization, GM and WM regions exhibited divergent age‐related trends in diffusion–relaxation MD‐MRI parameters. All regions showed positive linear age associations with isotropic diffusivity, *E*[$D_{\mathrm{iso}}$]; however, significant quadratic associations, after correction for multiple comparisons, were restricted to GM regions (Figure 4). Diffusion anisotropy, *E*[$D_{∆}^{2}$], showed positive linear age associations in GM and negative associations in WM, although most did not survive FDR correction. Transverse relaxation, *E*[$R_{2}$], exhibited weak negative linear age associations across most regions, while demonstrating strong inverted U‐shaped quadratic relationships in GM.

In addition to changes in average parameter values, variance measures exhibited strong and spatially extensive age effects. Variances of isotropic diffusivity and relaxation rates, particularly *V*[$D_{\mathrm{iso}}$] and *V*[$R_{2}$], increased linearly with age across association cortices, limbic regions, deep nuclei, and major WM tracts (Figure 4). These findings indicate increasing intra‐voxel heterogeneity and a broader dispersion of diffusion–relaxation environments in older adults, extending beyond shifts in average tissue properties.

Strong negative linear associations with age were observed for the diffusion frequency dependence of isotropic diffusivity, ${∆}_{\omega /2\pi }$ *E*[$D_{\mathrm{iso}}$], across all regions (Figure 4). These findings suggest a progressive reduction in restriction‐driven frequency dependence with age, consistent with decreased microstructural complexity and greater extracellular water contributions.

Estimation precision for all age effects is reported as 95% CI width in Figure S9; ROI‐parameter pairs with wide CIs should be interpreted with caution pending replication in larger cohorts.

### 3.6 Linear and Nonlinear Age Associations in Microstructural Phenotypes

Rather than tracking changes in mean parameter values, we examine here how the relative prevalence of specific microenvironmental phenotypes, jointly defined by diffusion and relaxation properties, redistributes with age across cortical, deep GM, and WM ROIs (Figure 5). For each region, regression models evaluated linear ($\beta_{\mathrm{age}}$) and quadratic ($\beta_{{\mathrm{age}}^{2}}$) age effects while controlling for sex, with significance assessed using false‐discovery‐rate correction. The resulting z‐scored coefficients indicate per‐year changes in phenotype in standard deviation units. Because these microstructural phenotypes represent normalized signal fractions that sum to one, they are inherently interconnected, and their age trajectories are best interpreted together.

**FIGURE 5:**
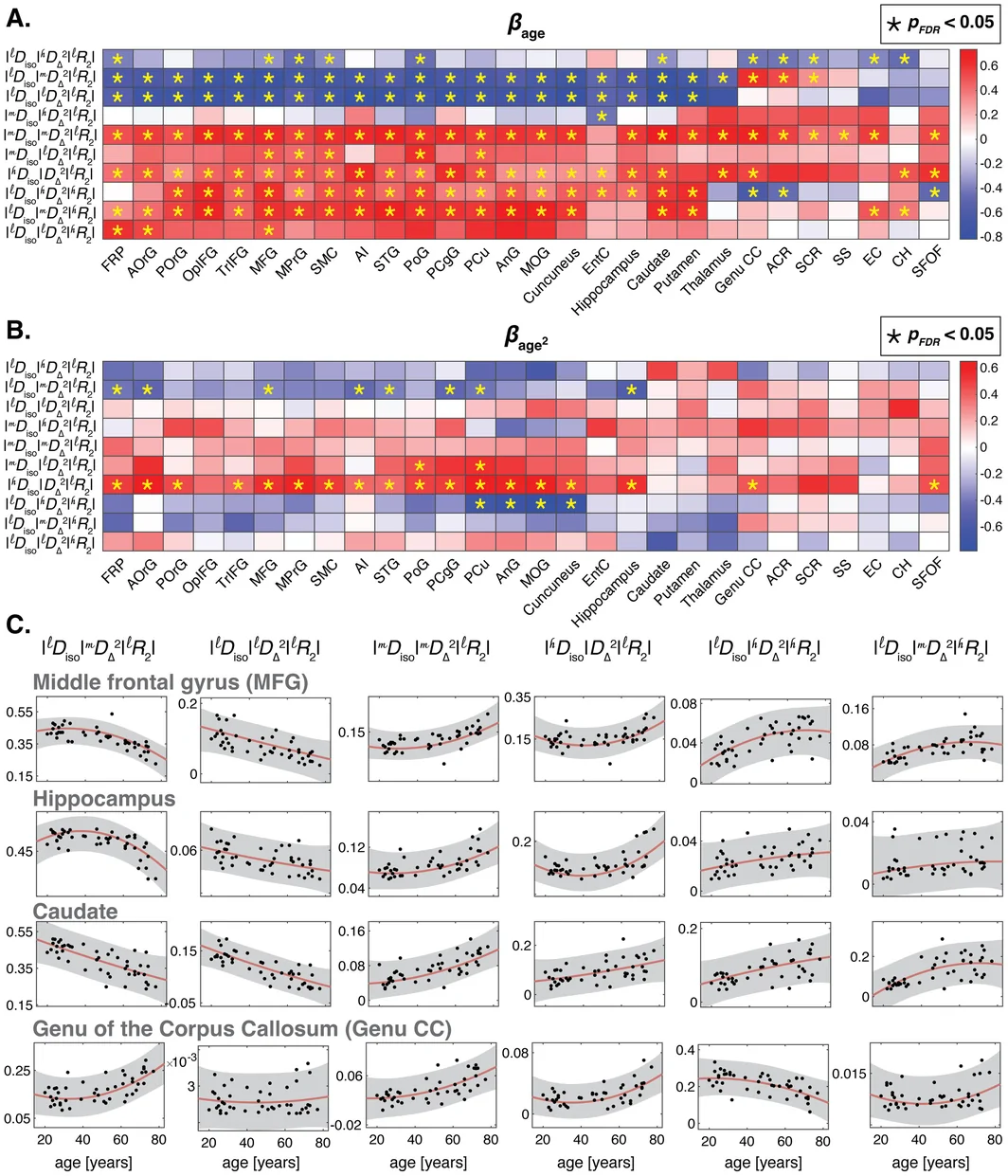
Linear and nonlinear age associations of microstructural phenotypes across brain regions. (A) Standardized linear age coefficients ($\beta_{\mathrm{age}}$) estimated for signal fractions associated with distinct diffusion–relaxation subdomains across cortical, deep GM, and WM ROIs. (B) Corresponding quadratic age coefficients ($\beta_{{\mathrm{age}}^{2}}$) capturing nonlinear lifespan trajectories. Scatter plots from representative regions and MD‐MRI parameters are shown in panel (C). Solid lines denote model fits, with shaded regions indicating 95% confidence intervals. Phenotypes with ROI‐averaged intensity under 0.01 were omitted from the plot to avoid bias.

When examining the low $R_{2}$ subdomains, GM regions generally exhibited age‐related decreases in phenotypes characterized by small cellular length‐scales (i.e., 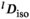), with corresponding increases in phenotypes associated with intermediate and large length‐scales (i.e., 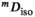 and 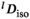). Only two phenotypes, |
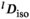
|
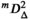
|
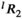
| and |
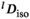
|$D_{∆}^{2}$|
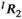
|, exhibited strong and consistent quadratic age relationships (Figure 5A,B). Microstructurally, these observations are consistent with decreased presence of cellular barriers and greater extracellular water contributions with age.

In WM, redistribution is limited in low $R_{2}$ subdomains, with negative linear associations in highly anisotropic, small length‐scale phenotypes and positive associations in intermediate‐anisotropy and unrestricted, high‐diffusivity phenotypes. Accordingly, |
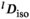
|
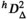
|
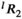
| shifts toward |
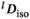
|
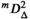
|^l^ 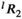
| and |
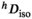
|$D_{∆}^{2}$|
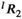
|. From a microstructural perspective, these findings reflect reduced cellular barriers, increased orientational dispersion, and greater extracellular water contributions with aging.

Finally, we examined the high $R_{2}$ subdomains, which are sensitive to macromolecular and iron content. Several age‐related patterns emerged. First, all high $R_{2}$ phenotypes were characterized by small cellular length scales. Second, significant positive linear age associations were confined to high‐ and intermediate‐anisotropy phenotypes and were observed primarily in GM, consistent with iron accumulation. Third, negative linear age associations were detected in the genu of the corpus callosum, anterior corona radiata, and superior fronto‐occipital fasciculus, consistent with myelin loss.

As with the diffusion‐relaxation parameters, 95% CI widths for all age effects are provided in Figure S10. In addition, these age associations were robust to the choice of subdomain thresholds: across all 243 threshold configurations, 93.6% of the phenotype–ROI associations significant in the main analysis remained significant, the sign of *β*_age_ was preserved in 99.8% of cases, and the coefficient of variation of *β*_age_ was 0.09 on average (Figure S11).

### 3.7 Diffusion Frequency Dependence of Microstructural Phenotypes

Up to this point, all reported microstructural phenotypes were evaluated at the lowest diffusion frequency. Diffusion frequency dependence has not previously been examined in the context of brain aging; the analysis presented here therefore represents a first characterization of how restriction‐driven frequency sensitivity varies across microstructural phenotypes and age groups. Because time‐ or frequency‐dependent diffusion arises from microscopic restriction imposed by cellular membranes, it provides mechanistic interpretability that complements the phenotype redistribution findings above. In the following analysis, we explicitly examine the dependence of microstructural phenotypes on diffusion frequency, reporting differences between the highest ($\omega_{\max}$ = 21 Hz) and lowest ($\omega_{\min}$ = 6.6 Hz) frequencies.

Figure 6 summarizes the diffusion frequency dependence of the microstructural phenotypes. Panels A and B show the absolute and relative differences, respectively, between high‐ and low‐frequency estimates averaged over the young adult group. Differences are separated and color‐coded by brain region (cortical, subcortical, and WM).

**FIGURE 6:**
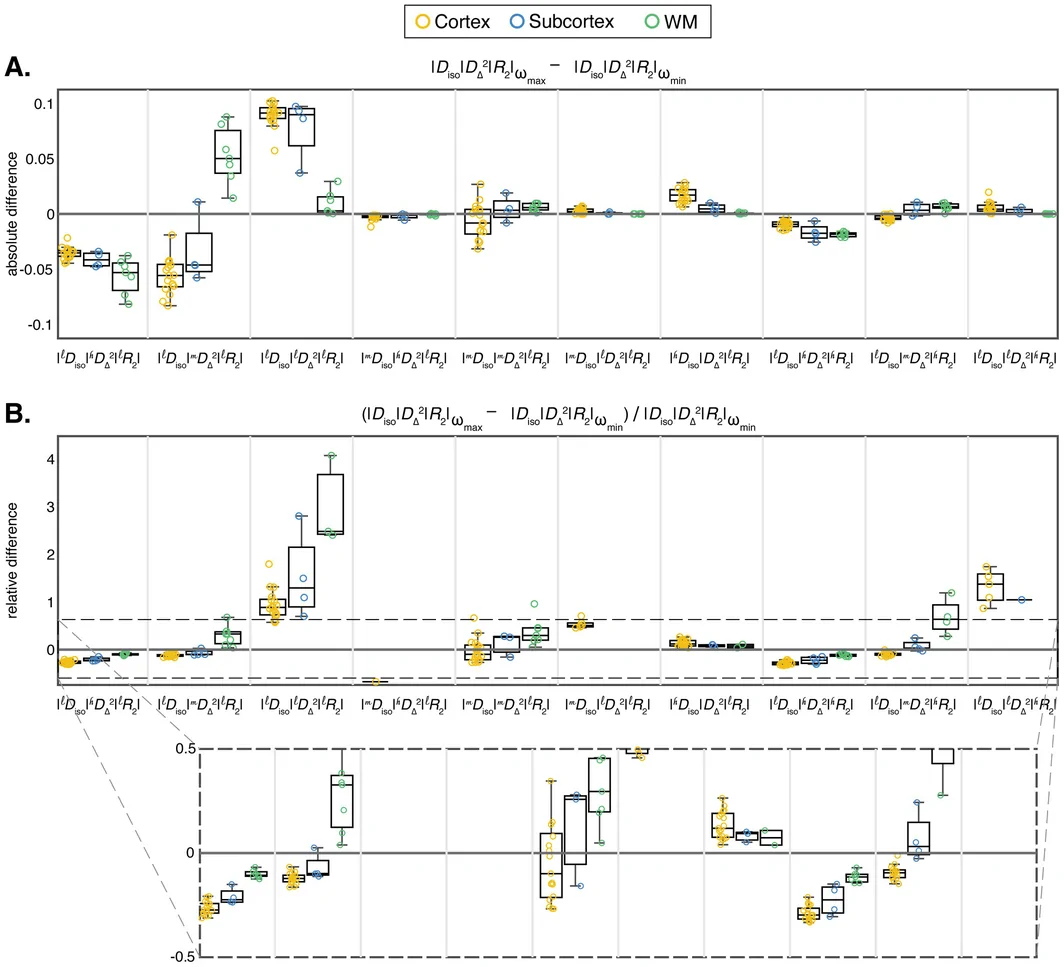
Diffusion frequency dependence of microstructural phenotypes. Panels (A and B) show the absolute and relative differences, respectively, between high‐ and low‐frequency estimates averaged across the young adult group. Differences are grouped and color‐coded by brain region (cortical GM, subcortical GM, and WM). Phenotypes with ROI‐averaged intensity under 0.01 were omitted from the plot to avoid bias.

Considerable frequency dependence was observed in several microstructural phenotypes. The biggest effect was observed in the near‐isotropic, small length‐scale parameters, namely, |
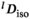
|
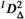
|
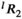
| and |
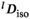
|
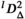
|
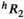
|. In both cases, higher diffusion frequencies are associated with larger signal fractions (up to 300% difference in WM, Figure 6B). The high anisotropy, small length‐scale parameters, |
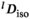
|
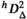
|
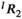
| and |
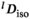
|
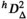
|
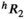
|, also exhibited strong restriction‐driven frequency dependence. Meanwhile, the large length‐scale parameter, |
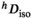
|$D_{∆}^{2}$|
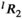
|, showed very little relative change with frequency, pointing to a lack of restriction.

The corresponding analysis for the aged adult group is provided in the Supporting Information S1 (Figure S11), illustrating absolute and relative differences between high‐ and low‐frequency estimates and confirming the presence of diffusion frequency–dependent effects. The age associations of the relative differences parameter are shown in Figure S12.

## 4 Discussion

Using MD‐MRI, this cross‐sectional study mapped novel microstructural phenotypes in a cohort of cognitively unimpaired adults spanning the full adult lifespan and characterized age‐related multilateral shifts within a joint diffusion–relaxation landscape that prior b‐tensor and relaxometry studies have not been able to resolve jointly. Rather than focusing on isolated diffusion or relaxation metrics, the MD‐MRI framework resolves continuous voxel‐wise distributions in a joint diffusion–relaxation space, enabling an integrated description of how cellular shape, size, restriction, and chemical composition reorganize with age. Our results indicate that cellular‐scale shape and restriction play an important role in brain aging, with small length‐scale structures exhibiting near‐isotropic or intermediate anisotropy remodeling into larger length‐scale structures. In parallel, fast‐relaxing *R*_2_ phenotypes exhibiting cellular‐scale restriction increase with age in GM and decrease in WM, likely reflecting iron accumulation and myelin loss, respectively.

Popular biophysical compartment models such as SANDI and NODDI assign parameters to predefined cellular structures by assuming a simplified microstructural model a priori and then fitting the data to estimate the relative contributions of each compartment. In contrast, MD‐MRI inverts this paradigm by first reconstructing continuous diffusion–relaxation distributions directly from the measured signal, without imposing assumptions about compartment number or tissue geometry, and subsequently deriving empirical “phenotypes” that quantify signal contributions from specific subdomains of the joint diffusion–relaxation parameter space.

Prior MRI studies reported microstructural simplification in the aging brain, including increased diffusivity, reduced anisotropy, and altered relaxation properties across WM regions (Qian et al. 2020; Schilling et al. 2022). Recent studies using b‐tensor encoding have further shown that microscopic fractional anisotropy decreases with age in white matter of older adults, and that this reduction underlies the kurtosis reductions previously reported in aging and Parkinson's disease (Kamiya et al. 2020; Olsson et al. 2025). Recent GM‐focused diffusion studies similarly found decreased cellular density and increased microstructural heterogeneity with age (Bouhrara et al. 2023; Lee et al. 2024; Singh et al. 2024, 2025). These results can be interpreted as different projections of a common underlying redistribution of diffusion–relaxation parameters, each capturing one dimension of a higher‐dimensional microstructural landscape. Extending this perspective, we show here that aging is marked by coordinated shifts within a joint diffusion–relaxation parameter space (Figure 1), rather than by the appearance of distinct new signal components. The diffusion–relaxation phenotypes identified here therefore provide a compact and interpretable summary of dominant signal states, without implying a one‐to‐one correspondence with specific cellular or histological compartments.

The age‐related patterns observed in conventional diffusion and relaxation parameters (Figures 2 and 4) are consistent with prior reports. In particular, the strong quadratic U‐shaped association between age and *E*[$D_{\mathrm{iso}}$], comparable to the DTI‐derived mean diffusivity, has been previously described (Bouhrara et al. 2023; Singh et al. 2024). Similarly, an inverted U‐shaped age dependence of E[$R_{2}$] has also been reported (Hagiwara et al. 2021). While conventional diffusion and relaxation MRI parameters have been extensively investigated in the context of aging, MD‐MRI uniquely enables the characterization of intra‐voxel variance. Beyond changes in mean parameter values, the observed age‐related increases in variance across diffusion and relaxation metrics suggest a progressive rise in intra‐voxel microstructural heterogeneity with aging, which could be driven by changes in extracellular volume and the presence of reactive astrocytes and microglia. The prominence of these effects in frontal and parietal association cortices points to an increasing dispersion of cellular and tissue environments in regions known to be particularly vulnerable to age‐related reorganization (Resnick et al. 2003). While these mean parameter and variance findings build on and extend the prior diffusion and relaxometry literature, the primary novel contribution of the present study lies in the phenotype redistribution analysis described below—which characterizes aging not through changes in individual parameters but through the reorganization of specific microenvironmental states defined by the co‐occurrence of diffusion and relaxation properties.

Another distinctive feature of this study is the ability to characterize the frequency dependence of *E*[$D_{\mathrm{iso}}$] and *E*[$D_{∆}^{2}$]. Because diffusion frequency dependence is directly linked to microscopic restriction (Yon et al. 2025), the observed age‐related reductions in ${∆}_{\omega /2\pi }$ *E*[$D_{\mathrm{iso}}$] together with predominantly positive linear and quadratic age associations in ${∆}_{\omega /2\pi }$ *E*[$D_{∆}^{2}$] across cortical, subcortical, and WM regions point to a systematic alteration of cellular‐scale barriers with aging. Collectively, these patterns are consistent with a progressive breakdown of microstructural restriction and with expansion of the extracellular space, reflecting age‐related weakening or reorganization of cellular boundaries rather than uniform changes in bulk diffusivity.

The primary novel contribution of this study—and what distinguishes it from prior b‐tensor aging work (Kamiya et al. 2020; Olsson et al. 2025) and from the relaxometry literature (MacDonald and Pike 2021)—is the characterization of aging as a redistribution of signal fractions across specific microenvironmental states defined by the *simultaneous* co‐occurrence of diffusion and relaxation properties. Prior b‐tensor studies tracked mean microscopic anisotropy; prior relaxometry studies tracked mean *T*_1_, *T*_2_, or *T*_2_* in isolation; the present framework instead asks which phenotypes, jointly characterized by their diffusion length, shape, and chemical environment, gain or lose prevalence with age, and whether these shifts are coherent across the brain. To this end, we defined in this study 10 subdomains in the joint $D_{\mathrm{iso}}$–$D_{∆}^{2}$–$R_{2}$ space, which capture different aspects of cellular‐level morphology and chemical environment. The resulting maps, shown in Figure 3, quantify the normalized signal fraction from each of the subdomains and, importantly, sum to 1. Age‐related redistributions within low‐$R_{2}$ diffusion–relaxation subdomains point to systematic reorganization of cellular‐scale microstructure with aging (Figure 5). In GM, the shift away from phenotypes associated with small cellular length scales toward intermediate and larger length‐scale components can be hypothesized to reflect a reduction in effective cellular barriers and an increased contribution of extracellular water. The presence of quadratic age relationships in select phenotypes further suggests non‐monotonic remodeling processes rather than simple linear degradation. In WM, redistribution within low‐$R_{2}$ subdomains was more constrained but followed a coherent pattern, with decreases in highly anisotropic, small–length‐scale phenotypes accompanied by increases in intermediate‐anisotropy and less restricted components. This pattern is indicative of increased orientational dispersion and extracellular expansion, consistent with age‐related loosening of microstructural organization rather than uniform loss of fiber architecture.

Distinct age‐related effects emerged within high‐$R_{2}$ subdomains, which are sensitive to macromolecular density and iron content. The restriction of high‐$R_{2}$ phenotypes to small cellular length scales across tissues suggests that these signal components predominantly reflect compact microstructural environments. Positive age associations observed primarily in anisotropic GM phenotypes are consistent with progressive iron accumulation (Burgetova et al. 2021), whereas negative age associations localized to anterior WM tracts, including the genu of the corpus callosum and corona radiata, align with known patterns of age‐related myelin loss (Qian et al. 2020). The directional pattern of these relaxation changes is consistent with the extensive relaxometry literature reviewed by (MacDonald and Pike 2021); the contribution here is to identify *which diffusion microenvironments* the relaxation changes co‐occur with—specifically, that the iron signal is concentrated in compact, anisotropic microenvironments rather than constituting a bulk tissue change. Together, these findings indicate that high‐$R_{2}$ subdomains capture tissue‐specific aging processes that are distinct from the extracellular and barrier‐related changes observed in low‐$R_{2}$ phenotypes.

The strong diffusion frequency dependence observed across specific diffusion–relaxation phenotypes provides insight into age‐related changes in microstructural restriction imposed by cellular membranes and barriers. The pronounced frequency sensitivity of near‐isotropic, small length‐scale phenotypes (Figure 6) suggests that these signal components arise from water pools confined by dense arrangements of cellular membranes and other restricting structures, whose contribution becomes more prominent when diffusion probes shorter length‐ and time‐scales. The magnitude of these effects, particularly in WM, highlights the value of frequency‐dependent measurements for capturing age‐related alterations in the prevalence and organization of restricting barriers. Similarly, the frequency dependence observed in highly anisotropic, small‐length‐scale phenotypes is consistent with restriction within elongated, membrane‐bounded structures such as axons or glial processes. In contrast, the relative frequency insensitivity of large length‐scale, high‐diffusivity phenotypes supports their interpretation as reflecting weakly restricted or extracellular environments, which are expected to contribute more prominently as restricting cellular barriers become less dominant with aging. Together, these findings indicate that diffusion frequency encoding provides a sensitive means of probing age‐related reorganization of membrane‐associated microstructural barriers and of extracellular volume.

The coordinated redistribution of diffusion–relaxation signal states observed here is consistent with multiple, partially overlapping biological processes known to accompany normative brain aging. Histological and ultrastructural studies have documented age‐related dendritic regression (Dickstein et al. 2013), synaptic pruning (Morrison and Baxter 2012), and glial remodeling across cortical and subcortical regions (Popov et al. 2021), together with gradual expansion of the extracellular space, all of which are expected to reduce microscopic restriction and directional coherence. Within this context, age‐related shifts in WM—including changes in microscopic diffusion anisotropy, attenuation of diffusion‐frequency dependence, and alterations in high‐$R_{2}$ phenotypes—can be hypothesized to reflect reorganization of axonal packing, myelin‐associated structure, and glial contributions. In cortical GM, the broadening of diffusion–relaxation distributions and the expansion of intermediate‐ and high‐diffusivity subdomains can be interpreted as increasing heterogeneity of the neuropil, where dendritic simplification, synapse loss, astrocytic remodeling, and extracellular expansion may coexist within individual voxels. Deep GM regions exhibited a distinct aging signature marked by increased high‐$R_{2}$ phenotype prevalence and reduced microscopic diffusion anisotropy; given the complex architecture of these regions, including the presence of crossing fibers, these changes are consistent with reduced neurite orientation dispersion and a decline in crossing fiber contributions, with potential implications for regional connectivity.

Several limitations should be considered when interpreting these findings. First, the cross‐sectional design limits inference about within‐subject aging trajectories and cannot fully separate aging effects from cohort‐related influences. Second, the signal model assumes non‐exchanging compartments. Although the estimated frequency dependence may reflect contributions from both restriction and exchange within the sampled frequency range, these mechanisms cannot be disentangled within the present framework, which may be particularly relevant in GM (Chakwizira, Westin, et al. 2023; Chakwizira, Zhu, et al. 2023; Williamson et al. 2019). Moreover, microstructurally distinct environments with characteristic length scales outside the sensitivity range of the diffusion encoding may not be separable in the recovered distributions. Consistent with previous work (Nilsson et al. 2017), the resulting diffusion–relaxation phenotypes should therefore be interpreted as empirical signal states rather than unique cellular compartments. Third, the relatively narrow frequency range of 6.6–21 Hz limits sensitivity to restricted diffusion. The asymmetric gradient waveforms are also susceptible in principle to concomitant‐field bias (Baron et al. 2012; Szczepankiewicz et al. 2019). Because the frequency‐dependence maps exhibit an anterior–posterior gradient that could resemble such bias, we quantified this effect for the waveforms and acquisition parameters used here. The predicted bias was small, spatially inconsistent with the observed pattern, and had little effect on the fitted parameter in a voxel‐wise perturbation analysis (see Supporting Information S1). Finally, the present analyzes were restricted to cognitively unimpaired adults, limiting generalizability to pathological aging populations.

Longitudinal MD‐MRI studies will be necessary to establish the temporal progression and rate of microstructural redistribution. Definitive attribution of the identified phenotypes to specific cellular or histological structures will require integration with complementary imaging modalities and postmortem validation. Further optimization of the frequency encoding range (Yon et al. 2025) and inversion strategies (Park et al. 2026) may improve sensitivity to restriction, and future implementations incorporating simultaneous multi‐slice acceleration are expected to substantially reduce acquisition time. Maxwell‐compensated waveform designs (Szczepankiewicz et al. 2019) should be considered to mitigate concomitant field effects. Extending this framework to pathological aging and neurodegenerative populations will be essential to determine the specificity and clinical relevance of diffusion–relaxation phenotypes, as will investigating associations between the identified phenotypes and cognitive performance.

## 5 Conclusion

Using MD‐MRI, we demonstrate that normative brain aging is characterized by coordinated redistributions within a joint diffusion–relaxation parameter space, rather than by the emergence of discrete new microstructural features. This multidimensional framework reveals age‐related increases in intra‐voxel heterogeneity alongside phenotype‐specific shifts, including a redistribution from small to larger effective length scales, reduced signatures of microscopic restriction, and tissue‐dependent changes in fast‐relaxing signal components. Together, these patterns are consistent with progressive reorganization of cellular‐scale barriers, increased extracellular contributions, and distinct gray‐ and white‐matter aging processes, including iron accumulation and myelin loss. By resolving diffusion–relaxation features and incorporating diffusion‐frequency dependence, MD‐MRI provides an integrated, model‐free description of how cellular‐scale size, shape, restriction, and chemical environment reorganize with age, complementing conventional diffusion and relaxometry measures. Although longitudinal and multimodal validation will be required to establish biological specificity, these findings position multidimensional diffusion–relaxation phenotyping as a promising framework for future studies linking microstructural reorganization to age‐related functional and cognitive decline.

## Author Contributions

Study design: D.B., Y.Y. Study conduct and data collection: J.S.P., Y.Y., D.B. Data analysis: J.S.P., E.M., S.B., B.A.L., D.B. Data interpretation: J.S.P., D.B. Drafting the manuscript: J.S.P., D.B.

## Funding

This work was supported by the National Institute on Aging.

## Conflicts of Interest

The authors declare no conflicts of interest.

## Supporting information


**Table S1:** Comprehensive summary of the in vivo MD‐MRI acquisition protocol.
**Table S2:** Ground truth simulation MD‐MRI components.
**Figure S1:** MD‐MRI acquisition and encoding design. (A) Time‐dependent optimized diffusion gradient waveforms *G*_x_(*t*), *G*_y_(*t*), and *G*_z_(*t*) used to generate linear, planar, and spherical b‐tensors at different echo times. (B) Corresponding diffusion spectra *b*(*ω*), illustrating the broad frequency content of the encoding waveforms. (C) Acquisition protocol showing the repetition time (TR), echo time (TE), b‐tensor magnitude (*b*), normalized anisotropy (*b*_Δ_; planar: −0.5, spherical: 0, linear: 1), gradient orientation angles (*θ*, *ϕ*), and centroid frequency *ω*_cent_/2*π*, plotted as a function of acquisition index.
**Figure S2:** Spatial comparison of predicted concomitant‐field bias and observed diffusion frequency dependency. (A) Axial slice of the observed Δ_ω/2π_E[*D*_iso_] map. Markers indicate the observed parameter peak (*Y* = −55 mm) and the predicted concomitant‐field bias peak (*Y* = +65 mm), located on opposite anterior–posterior sides of isocenter. (B) Anterior–posterior profiles of the observed parameter (blue, right axis) and protocol‐averaged predicted bias (green, left axis), averaged over *X* and *Z* within the brain mask.
**Figure S3:** Voxel‐wise propagation of predicted concomitant‐field bias into diffusion frequency dependency estimations. At each brain voxel, synthetic signals for all 139 waveforms were generated from the fitted parameters, multiplied by the predicted concomitant‐field attenuation, and refit. Left: unperturbed Δ_ω/2π_E[*D*_iso_] map. Center: perturbed map. Right: voxel‐wise difference (note the 10× finer color scale).
**Figure S4:** Grouped boxplots showing the relative bias (%) of each output parameter across 900 noise realizations at three SNR levels (green: high SNR = 542.3; blue: median SNR = 298.5; red: low SNR = 157.9). For each parameter group, boxes show the interquartile range, the horizontal line indicates the median, and whiskers extend to the 5th and 95th percentiles. The dashed horizontal line indicates zero bias. Bias below 15% (gray shaded region) was considered acceptable given the complexity of the forward model and the single canonical ground truth spectrum used. The two parameters exceeding this threshold ($V\left[D_{\Delta }^{2}\right],\Delta_{\omega /2\pi }E\left[D_{\Delta }^{2}\right]$) are both related to diffusion anisotropy and their elevated bias is attributable to gradient hardware limitations of clinical MRI scanners rather than noise floor effects.
**Figure S5:** Each cell shows the mean relative bias (%) for one parameter (rows) at one SNR level (columns). The color scale is symmetric around zero: blue indicates positive bias (overestimation), red indicates negative bias (underestimation), and white indicates no bias. SNR levels correspond to the 5th percentile, median, and 95th percentile of the per‐ROI median SNR distribution observed across subjects and ROIs (SNR = 157.9, 298.5, and 542.3 respectively). The pattern of bias is consistent across SNR levels for all parameters, confirming that estimation errors are driven by model complexity rather than by noise floor effects at the SNR levels achieved in this study.
**Figure S6:** Age‐related redistribution of joint diffusion‐relaxation phenotypes across major brain tissue classes. Group‐averaged three‐dimensional joint $D_{\mathrm{iso}}-D_{\Delta }^{2}-R_{2}$ distributions projected at maximal frequency, *ω*_min_ = 21 Hz, are shown for young (left column) and aged adults (middle column) in the (A) genu of the corpus callosum, (B) middle frontal gyrus (MFG), and (C) putamen. Nested isosurfaces are shown at the 98th, 99th, 99.5th, and 99.9th percentiles of the grand‐mean distribution pooled across all subjects, colored from deep blue (lowest) to red (highest) with opacity increasing cubically with percentile level. On the right column, red (positive) and blue (negative) isosurfaces show regions of difference between aged and young adults, rendered at four opacity levels spanning 50–100% of the peak absolute difference. White surface indicates zero. Across brain regions, aging is marked by multilateral microstructural shifts that reflect complex trajectories and together form a shared aging signature.
**Figure S7:** Region‐of‐interest–averaged MD‐MRI diffusion–relaxation parameters, stratified by age group.
**Figure S8:** Region‐of‐interest–averaged MD‐MRI microstructural phenotypes, stratified by age group. Some phenotypes have clear regional specificity; for example, both 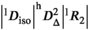 and 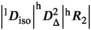 are more prevalent in WM. Conversely, lower anisotropy subdomains, e.g., 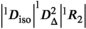 or 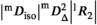, mainly dominate cortical and subcortical regions.
**Figure S9:** CI width heatmaps for the (A) linear (*β*_age_) and (B) quadratic $\left(\beta_{{\mathrm{age}}^{2}}\right)$ age regression coefficients across 28 ROIs (columns) and 10 diffusion‐relaxation parameters (rows). Color reflects the width of the 95% confidence interval, where lighter colors indicate greater estimation uncertainty. Asterisks denote ROI‐parameter pairs reaching statistical significance after FDR correction. CI width pattern is consistent across both age terms, differing only in overall magnitude.
**Figure S10:** CI width heatmaps for the (A) linear (*β*_age_) and (B) quadratic $\left(\beta_{{\mathrm{age}}^{2}}\right)$ age regression coefficients across 28 ROIs (columns) and 10 microstructural phenotypes (rows). Color reflects the width of the 95% confidence interval, where lighter colors indicate greater estimation uncertainty. Asterisks denote ROI‐parameter pairs reaching statistical significance after FDR correction. CI width pattern is consistent across both age terms, differing only in overall magnitude. Black cells indicate ROI‐parameter pairs with no signal variance across subjects and were excluded from analysis.
**Figure S11:** Robustness of age‐related microstructural effects to threshold perturbation. To assess the sensitivity of our findings to the heuristic boundaries used to define the microstructural phenotypes, we systematically perturbed each of the five internal threshold parameters (two on *D*_iso_: t1‐*D*_iso_, t2‐*D*_iso_; two on $D_{\Delta }^{2}$: $t1-D_{\Delta }^{2}$, $t2-D_{\Delta }^{2}$; one on *R*_2_: t‐*R*_2_) by −10%, 0%, or + 10%, yielding 3^5^ = 243 unique threshold configurations. For each configuration, the full analysis pipeline was rerun across all 46 subjects, and the quadratic regression model was recomputed for every phenotype–ROI pair. Each panel shows the effect of perturbing one threshold parameter, marginalizing over all combinations of the remaining four. Left axis (black): the fraction of bin/ROI pairs that were statistically significant in the main analysis (baseline, 0% shift; *p*_FDR_ < 0.05) that remained significant under perturbation. Right axis (orange): the mean relative change in the linear age coefficient, |Δ*β*_age_|/|*β*_age_|, averaged across all baseline‐significant bin/ROI pairs. Points represent individual threshold configurations at each shift level; error bars denote mean ± SD. Values near 1.0 (left) and near 0 (right) indicate that findings are robust to threshold choice.
**Figure S12:** Diffusion frequency dependence of microstructural phenotypes. Panels (A and B) show the absolute and relative differences, respectively, between high‐ and low‐frequency estimates averaged across the aged adult group. Differences are grouped and color‐coded by brain region (cortical GM, subcortical GM, and WM). Phenotypes with ROI‐averaged intensity under 0.01 were omitted from the plot to avoid bias.
**Figure S13:** Linear and nonlinear age associations of frequency dependence of microstructural phenotypes across brain regions. (A) Standardized linear age coefficients (*β*_age_) estimated for signal fraction frequency dependence associated with distinct diffusion‐relaxation subdomains across cortical, deep GM, and WM ROIs. (B) Corresponding quadratic age coefficients $\left(\beta_{{\mathrm{age}}^{2}}\right)$ capturing nonlinear lifespan trajectories. Phenotypes with ROI‐averaged intensity under 0.01 were omitted from the plot to avoid bias.

## Data Availability

Code to process the MD‐MRI data is freely available as implemented in the frequency dependent multi‐dimensional diffusion MRI toolbox (https://github.com/maximeYon/MMD). The data used in the study are available upon direct request as well as the conditions for its sharing or re‐use.
