# Supplementary material for: Multidimensional MRI Reveals Cellular‐Scale Microstructural Phenotypes in Human Brain Aging
Source: Aging Cell. 2026 Nov 3;25(11):e70782. doi: 10.1111/acel.70782 (PMC13731170; doi:10.1111/acel.70782)
Supplement: Supplementary file 1 [file ACEL-25-e70782-s001.pdf]

Supplementary Information: Multidimensional MRI reveals cellular-scale microstructural phenotypes in human brain aging

Joon Sik Park[1], Eppu Manninen[1], Shunxing Bao[2], Bennett A. Landman[2], Yihong Yang[3], Dan Benjamini[1,*]

[1] Multiscale Imaging and Integrative Biophysics Unit, National Institute on Aging, NIH, Baltimore, MD, USA

[2] Department of Electrical and Computer Engineering, Vanderbilt University, Nashville, TN, USA

[3] Neuroimaging Research Branch, National Institute on Drug Abuse, NIH, Baltimore, MD, USA

** Corresponding author: dan.benjamini@nih.gov*

Supplementary Methods

MD-MRI data acquisition

MRI system and pulse sequence: All MRI data were acquired on a 3T MAGNETOM Prisma system (Siemens Healthineers, Erlangen, Germany) using a 32-channel receive-only head coil. Multidimensional diffusion–relaxometry MRI (MD-MRI) data were collected using a single-shot spin-echo echo-planar imaging (EPI) sequence modified for tensor-valued diffusion encoding (TDE) with numerically optimized free gradient waveforms [1, 2].

Gradient waveforms were optimized using the md-grad toolbox (https://github.com/jan-martin-mri/md-grad), a MATLAB implementation of the constrained optimization scheme described by Sjölund et al. [3]. The optimization objective was to maximize the b-value for each encoding, subject to a maximum gradient amplitude of 80 mT/m and a maximum slew rate of 60 T/m/s. The scanner's hardware limit is 200 T/m/s; the reduced slew rate was chosen to limit gradient heating. Motion and flow compensation constraints were not applied. In frequency-dependent diffusion encoding, the signal attenuation is governed by the frequency-domain integral of the product of the dephasing power spectrum $\mathbf{b}(\omega)$–computed directly from the gradient waveform — and the diffusion spectrum $\mathbf{D}(\omega)$. Applying flow compensation, which nulls the first moment of the gradient waveform, redistributes spectral energy away from higher frequencies toward lower frequencies [4], which would systematically compress the effective frequency range of the encoding and reduce sensitivity to restriction at the higher end of the frequency window. Flow compensation was therefore deliberately avoided to preserve the integrity of the frequency-dependent diffusion measurement. Concomitant gradient field compensation (Maxwell compensation) was not applied; the resulting waveforms are therefore asymmetric with respect to the refocusing pulse, which introduces the potential for spatially varying signal bias as discussed in the Methods and addressed in the Discussion. A comprehensive overview of gradient waveform design considerations can be found in Szczepankiewicz et al. [5].

Acquisition parameters: MD-MRI was acquired at 2-mm isotropic spatial resolution with the following parameters: field of view (FOV) = $228 \times 228 \times 110$ mm^3, voxel size = $2 \times 2 \times 2$ mm^3, acquisition bandwidth = 1512 Hz/pixel, in-plane parallel imaging using GRAPPA with acceleration factor $R = 2$ (24 reference lines), effective echo spacing = 0.8 ms, partial Fourier factor = 0.75 in the phase-encoding direction, and axial slice orientation. A comprehensive summary of acquisition parameters is provided in supplementary Table 1.

Protocol design: The MD-MRI protocol comprised 139 unique diffusion–relaxation measurements with an overall scan time of approximately 40 minutes per participant. The protocol was derived from a previously reported 633-point $(D(\omega), R_1, R_2)$ acquisition [1],with encoding parameters empirically selected to reduce protocol dimensionality while preserving sensitivity to diffusion-relaxation contrasts and extending the ranges of repetition time (TR) and echo time (TE) relative to the original design.

Diffusion-relaxation encoding: Diffusion encoding was achieved using numerically optimized gradient waveforms [3] designed to generate linear ($b_\Delta = 1$), spherical ($b_\Delta = 0$), and planar ($b_\Delta = -0.5$) b-tensors, with b-values ranging from 0.1 to 3 ms/μm^2, in addition to one non-diffusion-weighted ($b = 0$ ms/μm^2) volume. Sensitivity to longitudinal and transverse relaxation was achieved by sampling multiple repetition times and echo times, with TR = (0.62, 1.75, 3.5, 5, 7, 7.6) s and TE = (40, 63, 83, 150) ms.

The diffusion gradient waveforms used in the acquisition and their corresponding diffusion spectra are shown in Supplementary Fig. S1. The applied waveforms exhibited broad spectral content characterized by centroid frequencies $\omega_{\mathrm{cent}}/2\pi$ spanning 6.6–21 Hz. Each waveform contains a distribution of frequencies, and the centroid frequency is reported only as a representative

descriptor. All subsequent signal modeling and inversion steps explicitly used the full spectral representation $b(\omega)$ rather than a single-frequency approximation. The maximum frequencies reached depended on the b-value, with approximate maxima of 21, 15, and 11 Hz for $b = 0.5$, 1.5, and 3 ms/μm^2, respectively.

To enable correction of susceptibility-induced geometric distortions, MD-MRI data were acquired with a single anterior-to-posterior (AP) phase-encoding direction together with an additional reversed posterior-to-anterior (PA) $b = 0$ volume.

Structural imaging: In addition to MD-MRI, fat-suppressed T_1-weighted MPRAGE images were acquired for anatomical reference and ROI definition (TR = 1900 ms, TE = 3.42 ms, 1-mm isotropic resolution). Structural images were used exclusively for image registration and anatomical segmentation.

Parameter	Value / Description
Scanner	3T MAGNETOM Prisma (Siemens Healthcare, Erlangen, Germany)
Head coil	32-channel receive-only
Structural scan	Fat-suppressed T_1-weighted MPRAGE (1 mm isotropic)
Sequence	Single-shot spin-echo EPI with tensor-valued diffusion encoding
Voxel size	2 mm isotropic
Field of view (FOV)	$228 \times 228 \times 110$ mm^3
Bandwidth	1512 Hz/Px
Parallel imaging	GRAPPA (R = 2), 24 reference lines
Echo spacing	0.8 ms
Partial Fourier	0.75 (phase-encoding direction)
Phase encoding	AP (plus one reversed PA $b = 0$ for distortion correction)
Repetition times (TR)	0.62, 1.75, 3.5, 5, 7, 7.6 s
Echo times (TE)	40, 63, 83, 150 ms
Diffusion weighting (b)	0.1–3 ms/μm^2
B-tensor shapes	Linear ($b_\Delta = 1$), Planar ($b_\Delta = -0.5$), Spherical ($b_\Delta = 0$)
Frequency range	Centroid $\omega_{\text{cent}}/2\pi = 6.6$–21 Hz
Number of volumes	139 unique diffusion-relaxation measurements
Scan time	40 minutes

Supplementary Table 1: Comprehensive summary of the in vivo MD-MRI acquisition protocol.

MD-MRI data processing

Data preprocessing: MD-MRI data were preprocessed following recent recommendations for sparse multidimensional diffusion–relaxometry MRI acquisitions [6]. All diffusion-weighted volumes were first concatenated and denoised using the Marchenko–Pastur principal component analysis (MP-PCA) method [7]. For partial Fourier acquisitions, Gibbs ringing correction was applied using a local subvoxel-shift approach [8, 9].

Motion- and eddy-current-induced distortions were corrected using the DIFFPREP module of the TORTOISE software package [10], employing a physically based parsimonious quadratic transformation model together with a normalized mutual information similarity metric. Susceptibility-induced geometric distortions were corrected using the DR-BUDDI framework [11]. For this purpose, the acquired anterior–posterior (AP) and posterior–anterior (PA) phase-encoding $b = 0$ images were combined with a synthetic T_2-weighted image generated from the T_1-weighted anatomical scan [12].

All preprocessing steps were combined into a single final interpolation step, yielding fully corrected MD-MRI volumes aligned to anatomical space at native MD-MRI resolution (2-mm isotropic). No visible slice-to-volume motion or motion-induced signal dropouts were observed in this dataset; therefore, these correction options were not applied.

Signal modeling and Monte Carlo inversion: Preprocessed MD-MRI data were analyzed in MATLAB (MathWorks, Natick, MA) using a Monte Carlo (MC) inversion framework implemented in the multidimensional diffusion MRI toolbox [13, 14, 15, 16]. The MD-MRI signal was modeled as a weighted superposition of discrete components spanning the joint diffusion-relaxation parameter space according to

$$S\left(\mathbf{b}(\omega), \text{TE}, \text{TR}\right) = \sum_{i=1}^{N_c} f_i \exp\left(-\int_{-\infty}^{\infty} \mathbf{b}(\omega) : \mathbf{D}_i(\omega)\, d\omega\right)\left(1 - e^{-\text{TR}\cdot R_{1,i}}\right) e^{-\text{TE}\cdot R_{2,i}}, \tag{1}$$

where f_i denotes the signal fraction of the i-th component and N_c is the number of fitted components.

Each frequency-dependent diffusion tensor $\mathbf{D}_i(\omega)$ was approximated as an axisymmetric Lorentzian parameterized by axial and radial diffusivities $(D_{\|,i}, D_{\perp,i})$, orientation angles (θ_i, ϕ_i), high-frequency isotropic diffusivity $D_{0,i}$, axial and radial transition frequencies $(\Gamma_{\|,i}, \Gamma_{\perp,i})$, and longitudinal and transverse relaxation rates $(R_{1,i}, R_{2,i})$ [17, 18, 19, 6]. These parameters were sampled within biologically plausible bounds: $0.05 \leq D_{\|}, D_{\perp}, D_0 \leq 5$ μm^2/ms; $0 \leq \theta \leq \pi$, $0 \leq \phi \leq 2\pi$; $0.2 \leq R_1 \leq 2$ s^{-1}, $1 \leq R_2 \leq 30$ s^{-1}; and $0.01 \leq \Gamma_{\|}, \Gamma_{\perp} \leq 10^4$ s^{-1}. Axial and radial diffusivities were reparameterized as isotropic diffusivity

$$D_{\text{iso}} = \frac{D_{\|} + 2D_{\perp}}{3}, \tag{2}$$

and squared microscopic diffusion anisotropy

$$D_{\Delta}^2 = \frac{(D_{\|} - D_{\perp})^2}{(D_{\|} + 2D_{\perp})^2}. \tag{3}$$

The MC inversion was performed using nonnegative least squares combined with quasi-genetic filtering and bootstrapping with replacement to address the ill-conditioned nature of the inverse problem [19, 15]. For each voxel, $N_b = 100$ bootstrap realizations were generated, each producing up to $N_c = 10$ discrete components. Algorithmic parameters were set to $N_{\text{in}} = 200$, $N_p = 20$, and $N_m = 1$. As a result, for each imaging voxel, a total of weighted signal components were estimated across bootstrap realizations, providing a robust empirical sampling of the underlying diffusion-relaxation signal. The resulting voxel-wise solutions were evaluated at selected values of $\omega/2\pi$ within the experimentally sampled frequency range (6.6-21 Hz), yielding frequency-dependent representations of the diffusion-relaxation signal in the $(D_{\text{iso}}(\omega), D_{\Delta}^2(\omega), R_1, R_2)$ space.

Following conventions often used to display results from oscillating gradient encoding the effects of restricted diffusion were quantified by a finite difference approximation of the rate of change of the diffusivity metrics with frequency within the investigated window, which in our case was $\omega_{\text{max}} = 21$ Hz and $\omega_{\text{min}} = 6.6$ Hz, and define

$$\Delta_{\omega/2\pi} E[D_{\text{iso}}] = \frac{E[D_{\text{iso}}(\omega_{\text{max}})] - E[D_{\text{iso}}(\omega_{\text{min}})]}{(\omega_{\text{max}} - \omega_{\text{min}})/2\pi}, \tag{4}$$

and

$$\Delta_{\omega/2\pi} E[D_{\Delta}^2] = \frac{E[D_{\Delta}^2(\omega_{\text{max}})] - E[D_{\Delta}^2(\omega_{\text{min}})]}{(\omega_{\text{max}} - \omega_{\text{min}})/2\pi}. \tag{5}$$

Diffusion-relaxation subdomains: The $(D_{\text{iso}}, D_{\Delta}^2, R_2)$ space was discretized into diffusion-relaxation subdomains defined by combinations of low, intermediate, and high regimes along each parameter dimension. Isotropic diffusivity was partitioned into three regimes corresponding to small, intermediate, and large microscopic length scales ($0.05 < D_{\text{iso}} < 1.5$, $1.5 < D_{\text{iso}} < 3$, and $D_{\text{iso}} > 3$ μm^2/ms, respectively). Squared microscopic diffusion anisotropy was likewise partitioned into low, intermediate, and high regimes ($0 < D_{\Delta}^2 < 0.01$, $0.01 < D_{\Delta}^2 < 0.25$, and $D_{\Delta}^2 > 0.25$), reflecting increasing degrees of microscopic directional constraint. Transverse relaxation was partitioned into two regimes, corresponding to slow-relaxing ($1 < R_2 < 20$ s^{-1}) and fast-relaxing ($R_2 > 20$ s^{-1}) signal components, which are sensitive to differences in macromolecular content and water-tissue interactions. For each voxel, the following signal fractions were computed by summing the weights of consolidated components within the corresponding diffusion-relaxation subdomains, which we term here "microstructural phenotypes":

1. $|^{\ell} D_{\text{iso}}|^{\hbar} D_{\Delta}^2|^{\ell} R_2|$: $0.05 < D_{\text{iso}} < 1.5$ μm^2/ms; $D_{\Delta}^2 > 0.25$; $1 < R_2 < 20$ s^{-1}.
2. $|^{\ell} D_{\text{iso}}|^{m} D_{\Delta}^2|^{\ell} R_2|$: $0.05 < D_{\text{iso}} < 1.5$ μm^2/ms; $0.01 < D_{\Delta}^2 < 0.25$; $1 < R_2 < 20$ s^{-1}.
3. $|^{\ell} D_{\text{iso}}|^{\ell} D_{\Delta}^2|^{\ell} R_2|$: $0.05 < D_{\text{iso}} < 1.5$ μm^2/ms; $0 < D_{\Delta}^2 < 0.01$; $1 < R_2 < 20$ s^{-1}.
4. $|^{m} D_{\text{iso}}|^{\hbar} D_{\Delta}^2|^{\ell} R_2|$: $1.5 < D_{\text{iso}} < 3$ μm^2/ms; $D_{\Delta}^2 > 0.25$; $1 < R_2 < 20$ s^{-1}.
5. $|^{m} D_{\text{iso}}|^{m} D_{\Delta}^2|^{\ell} R_2|$: $1.5 < D_{\text{iso}} < 3$ μm^2/ms; $0.01 < D_{\Delta}^2 < 0.25$; $1 < R_2 < 20$ s^{-1}.
6. $|^{m} D_{\text{iso}}|^{\ell} D_{\Delta}^2|^{\ell} R_2|$: $1.5 < D_{\text{iso}} < 3$ μm^2/ms; $0 < D_{\Delta}^2 < 0.01$; $1 < R_2 < 20$ s^{-1}.
7. $|^{\hbar} D_{\text{iso}}|D_{\Delta}^2|^{\ell} R_2|$: $D_{\text{iso}} > 3$ μm^2/ms; $0 < D_{\Delta}^2 < 1$; $1 < R_2 < 20$ s^{-1}.
8. $|^{\ell} D_{\text{iso}}|^{\hbar} D_{\Delta}^2|^{\hbar} R_2|$: $0.05 < D_{\text{iso}} < 1.5$ μm^2/ms; $D_{\Delta}^2 > 0.25$; $20 < R_2 < 30$ s^{-1}.
9. $|^{\ell} D_{\text{iso}}|^{m} D_{\Delta}^2|^{\hbar} R_2|$: $0.05 < D_{\text{iso}} < 1.5$ μm^2/ms; $0.01 < D_{\Delta}^2 < 0.25$; $20 < R_2 < 30$ s^{-1}.
10. $|^{\ell} D_{\text{iso}}|^{\ell} D_{\Delta}^2|^{\hbar} R_2|$: $0.05 < D_{\text{iso}} < 1.5$ μm^2/ms; $0 < D_{\Delta}^2 < 0.01$; $20 < R_2 < 30$ s^{-1}.

Data quality assessment

Head motion: Because diffusion-relaxation imaging acquisitions are relatively long and may be sensitive to participant motion, we performed both qualitative and quantitative assessments of data quality. Following preprocessing, all datasets were visually

inspected for residual motion artifacts, geometric distortions, image corruption, and preprocessing failures. No datasets were excluded on the basis of image quality.

Quantitative head-motion estimates were derived from the rigid-body transformations generated during the TORTOISE motion- and eddy-current-correction procedure. For each diffusion volume, TORTOISE estimates rigid-body translation and rotation parameters relative to a reference volume. Subject-level motion was summarized using the mean relative displacement between consecutive diffusion volumes. Relative displacement was calculated from volume-to-volume changes in the three translational and three rotational parameters, with rotational motion converted to linear displacement assuming a head radius of 50 mm. This approach yields a single composite measure of motion expressed in millimeters and is conceptually similar to the relative displacement metrics reported by diffusion MRI quality-control frameworks such as FSL EDDY and EDDY-QUAD [20] and to recent diffusion MRI studies that quantify motion using volume-to-volume displacement estimates derived from motion-correction parameters [21].

Across the cohort, mean relative displacement ranged from 0.24 to 0.85 mm. Thus, all participants exhibited average frame-to-frame motion substantially below the 2-mm isotropic voxel size used for data acquisition, indicating generally low motion levels throughout the study.

To determine whether motion differed systematically across age groups, mean relative displacement was compared using a Kruskal–Wallis test. A significant group effect was observed ($\epsilon^2 = 0.124$), and post hoc comparisons indicated greater motion in the oldest age group relative to the youngest age group. However, the magnitude of this difference was modest in absolute terms. Mean (median) relative displacement was 0.383 mm (0.350 mm), 0.408 mm (0.376 mm), and 0.492 mm (0.469 mm) for the young, middle, and aged groups, respectively. The difference between the youngest and oldest groups was approximately 0.11 mm, corresponding to about 5% of the voxel dimension.

Taken together, these analyses indicate that although motion increased modestly with age, overall motion levels remained low across the cohort and were substantially smaller than the acquired voxel size. Consequently, head motion is unlikely to be a major driver of the reported age-related diffusion-relaxation imaging findings.

Registration quality control: To assess whether registration quality differed systematically across age groups, we computed the log Jacobian determinant of the deformation field from each group-averaged diffusion anisotropy image to the JHU WM atlas, using ANTs' CreateJacobianDeterminantImage (geometric computation, log output). The log Jacobian determinant encodes local volume change induced by the warp: a value of zero indicates no deformation, positive values indicate expansion, and negative values indicate compression relative to the atlas. Statistics were extracted within a binary white matter mask derived from the JHU label atlas. The log Jacobian determinant was comparable across all three age groups (young: 0.044±0.345, middle: 0.054±0.307, aged: 0.023±0.315), with no systematic increase in deformation magnitude or variability in the aged group. These results argue against differential mislocalization of WM tract labels due to age-dependent registration bias.

Noise floor assessment: For each subject, the noise standard deviation σ was estimated from a background ROI containing no tissue signal. For each of the 139 measurements, the standard deviation of the signal across background voxels was computed. Because magnitude MRI signal in the absence of true signal follows a Rayleigh distribution, for which the standard deviation equals $\sigma(2-\pi/2)$, the per-measurement σ was obtained by dividing the background standard deviation by $(2-\pi/2) \approx 0.655$, and the 139 per-measurement estimates were averaged to yield a single σ per subject [22]. SNR was then computed per individual voxel as the ratio of the voxel signal to σ at each measurement. The median of these per-voxel SNR estimates across voxels within each ROI was taken as the representative ROI value. Importantly, this differs from computing SNR from the ROI-averaged signal: averaging signal across voxels suppresses noise and inflates SNR relative to what individual voxels experience during fitting. Since model fitting was performed voxel-wise, the per-voxel SNR distribution is the relevant quantity for assessing noise floor exposure.

Two SNR metrics were examined: SNR at the unweighted reference measurement (best case) and SNR at the most attenuated measurement across all 139 acquisitions (worst case). Since our analysis examines each ROI across all 46 subjects using regression, the relevant summary statistic is the median SNR across subjects within each ROI, rather than the global minimum across all subjects and ROIs. The latter is dominated by isolated subject–ROI outliers that contribute a single data point to the regression and are unlikely to influence group-level estimates.

At the unweighted reference measurement, the median SNR across subjects ranged from 153.8 to 556.9 across the 28 ROIs. At the most attenuated measurement, the median SNR across subjects ranged from 8.7 to 27.0 across the 28 ROIs. These values confirm that the typical subject in every ROI remained well above the noise floor throughout the acquisition. Rician bias–which becomes substantial below SNR $\approx$ 2–3 [22, 23]–is therefore not expected to meaningfully affect the estimated model parameters

in any ROI.

Concomitant field bias: The tensor-valued encoding waveforms used in this study are temporally asymmetric about the refocusing pulse, a design choice that maximizes encoding efficiency but leaves them subject to bias from concomitant gradient fields [24, 25]. Because the $\Delta_{\omega/2\pi}\mathrm{E}[D_{\mathrm{iso}}]$ map (Fig. 2, second column from the right) exhibits an anterior-posterior gradient that superficially resembles patterns concomitant-field bias can produce, we quantified the predicted bias for our specific waveforms and evaluated its contribution to the observed pattern.

We implemented the framework of Baron *et al.* [24], generalized to accommodate rank-1/2/3 tensor-valued encoding waveforms, and applied it to our 139-waveform protocol at the acquisition parameters used in this study (3T, 2 mm axial slices, anterior-posterior phase-encode). Predicted signal loss was evaluated in native scanner space within the brain mask of the acquired volume for a representative subject; because concomitant-field bias is determined by scanner geometry, waveform design, and FOV placement — not by subject-specific tissue properties — results are representative of the full cohort under comparable head positioning.

Across the protocol, peak voxel-wise signal loss reached 2.30% for the worst waveform (b = 1500 s/mm^2, at the superior-anterior periphery), with protocol-averaged mean signal loss of 0.11%. No waveform produced peak loss exceeding 5%, the threshold associate with meaningful bias in derived DTI metrics [24]. Predicted signal loss scaled with characteristic encoding frequency (Spearman $\rho = 0.64$, $p < 10^{-16}$; high-frequency quartile median 0.33%, low-frequency quartile 0.007%). This scaling does not indicate a direct effect of frequency, but reflects that higher-frequency waveforms in our protocol are also the most temporally asymmetric — asymmetry being the physical driver of the residual concomitant moment.

The spatial pattern of the protocol-averaged predicted bias differs from that of the observed $\Delta_{\omega/2\pi}\mathrm{E}[D_{\mathrm{iso}}]$ map (Fig. S2). The observed parameter peaks in the mid-posterior brain ($Y \approx -55$ mm from isocenter), while the predicted bias peaks at the anterior-superior periphery ($Y \approx +65$ mm) — on the opposite anterior-posterior side of isocenter. The predicted bias is dominated by the through-plane z^2 term of the concomitant field (voxel-wise correlation with $|z|$: $\rho = 0.98$). Voxel-wise spatial Spearman correlation between predicted bias and observed $\Delta_{\omega/2\pi}\mathrm{E}[D_{\mathrm{iso}}]$ is $\rho = +0.29$, weak in magnitude.

To quantify how the predicted signal-level bias propagates into $\Delta_{\omega/2\pi}\mathrm{E}[D_{\mathrm{iso}}]$ estimation through the fitting pipeline, we performed a voxel-wise propagation on the same subject. At each brain voxel, synthetic signals for all 139 waveforms were generated from the fitted parameters ("model"), multiplied by the predicted concomitant-field attenuation at that voxel and waveform, and refit ("perturbed"). The perturbed $\Delta_{\omega/2\pi}\mathrm{E}[D_{\mathrm{iso}}]$ map (Fig. S3, center) remains very similar to the unperturbed map (left). The voxel-wise difference (right) reaches a maximum value of $\sim 4 \times 10^{-12}$, an order of magnitude below the observed dynamic range ($\sim 4 \times 10^{-11}$), and lacks coherent spatial structure.

We conclude that concomitant-field bias does not account for the reported A-P pattern in $\Delta_{\omega/2\pi}\mathrm{E}[D_{\mathrm{iso}}]$ or its association with age at our acquisition parameters. This conclusion is specific to our acquisition parameters and imaging volume. For future studies employing frequency-dependent tensor-valued encoding, Maxwell-compensated waveform designs [25] eliminate this concern by construction.

Simulations

To assess the impact of noise on parameter estimation, simulations were performed using the forward model (Eq. 1 of the main text) with a single canonical ground truth (GT) spectrum representative of brain tissue (Table 2), as previously done [26, 27]. Synthetic signals were generated for all 139 measurements and Rician noise was added at three SNR levels corresponding to the 5th percentile, median, and 95th percentile of the per-ROI median SNR distribution observed across subjects and ROIs (SNR = 157.9, 298.5, and 542.3 at the unweighted reference measurement, respectively). These values bracket the full range of noise conditions in the study.

For each SNR level, 900 independent noise realizations were generated, simulating the number of voxels in a typical ROI. The full inversion pipeline was applied to each realization, and the recovered parameters were compared to ground truth. For each parameter X, bias was quantified as the median relative difference across 900 realizations: $bias(\%) = 100 \times median(X - X_{GT}/X_{GT})$.

Results are shown in Fig. S4 (distributions across realizations) and Fig. S5 (summary heatmap across parameters and SNR levels). Across the three SNR levels, median relative bias was below 15% for 11 of 13 parameters, with the majority below 10%. Bias was stable across SNR levels for most parameters, confirming that the observed estimation errors reflect model complexity and behavior rather than noise floor effects–consistent with the empirical finding that all measurements remained well above SNR = 2 throughout the acquisition. The two parameters with elevated bias–$\mathrm{V}[D_\Delta^2]$, $\Delta_{\omega/2\pi}E[D_\Delta^2]$–are both related to diffusion anisotropy. Their bias is consistent with the known limitations of clinical MRI gradient hardware, which constrains the

Supplementary Table 2: Ground truth simulation MD-MRI components.

Comp. \ Parameter	$D_{\|\|}$ [ms/μm^2]	$D_\perp$ [ms/μm^2]	θ [°]	ϕ [°]	D_0 [ms/μm^2]	$\Gamma_{\|\|}$ [Rad/s]	$\Gamma_\perp$ [Rad/s]	R_1 [1/s]	R_2 [1/s]	f
**I**	1.0	0.6	0	0	3	250	200	1	10	1/3
**II**	2.8	0.05	-30	20	3	2000	130	1.5	15	1/3
**III**	3.3	3.3	0	0	3.3	10000	10000	0.3	6	1/3

encoding of anisotropy information in diffusion measurements and is independent of the noise floor. These parameters should be interpreted with caution in the context of clinical acquisitions.

Estimation precision of age effects

Given the large number of ROI-parameter comparisons relative to the cohort size (n=46), we report 95% confidence interval (CI) width as a per-comparison measure of estimation precision for both the diffusion-relaxation parameters and microstructural phenotypes (Figs. S9 and S10, respectively).

CI width reflects uncertainty in the effect estimate independent of its direction or magnitude. Narrow CIs indicate reliably estimated effects; wide CIs flag ROI-parameter pairs where conclusions should be drawn with caution. Because CI width is determined by the residual variance of the outcome–shared across all predictors within the same model–the spatial pattern of CI widths is consistent across the linear (β_{age}) and quadratic (β_{age^2}) age terms, differing only in overall magnitude. Cells reaching statistical significance after FDR correction are marked with an asterisk, allowing direct cross-referencing of effect reliability and significance.

Several ROI-parameter pairs exhibit wide CIs in both parameter sets, consistent with limited statistical power per comparison. Significant effects associated with narrow CIs represent the most reliable findings; significant effects associated with wide CIs should be considered preliminary and warrant replication in larger cohorts.

Sensitivity analysis for microstructural phenotype domain thresholds

The ten microstructural phenotypes analyzed in this study are defined by partitioning the three-dimensional distribution over D_{iso}, D_{Δ}^2, and R_2 into cuboid domains, with the boundaries of each sub-region set by heuristic thresholds. Specifically, the partition is governed by five internal thresholds: two along the D_{iso} axis (t1-D_{iso}, t2-D_{iso}), two along the D_{Δ}^2 axis (t1-D_{Δ}^2, t2-D_{Δ}^2), and one along the R_2 axis (t-R_2), which together define the cuboids whose partial integrals constitute the phenotypes. Because these threshold values were chosen heuristically, we performed a sensitivity analysis to evaluate whether our age-related findings depend on the specific boundary values selected.

We systematically perturbed each of the five thresholds by -10%, 0%, or +10% of its original value. Taking all combinations across the five thresholds yielded $3^5 = 243$ unique threshold configurations, including the original (unperturbed) configuration in which all five thresholds are shifted by 0%. We verified that, under all perturbations within this ±10% range, the ordering of thresholds along each axis was preserved (t1-D_{iso} < t2-D_{iso} and t1-D_{Δ}^2 < t2-D_{Δ}^2), so that no perturbation produced a degenerate or inverted partition. The outer boundaries of the distribution along each axis were held fixed. For each of the 243 configurations, we regenerated the phenotype maps for all 46 subjects and repeated the full regression analysis. As in the main analysis, for each phenotype–ROI pair we fit the quadratic age regression model, and extracted the linear age coefficient (β_{age}) and its associated p-value, and then correcting for multiple comparisons. We then quantified robustness using two complementary measures, evaluated only on the set of phenotype–ROI pairs that were statistically significant in the main (unperturbed) analysis (p_{FDR}<0.05). First, as a measure of inferential stability, we computed the fraction of these baseline-significant pairs that remained significant under each perturbed configuration. Second, as a measure of effect-size stability that does not depend on a significance threshold, we computed the relative change in the age coefficient, $|\Delta\beta_{age}|/|\beta_{age}|$, between each perturbed configuration and the baseline, averaged across all baseline-significant pairs.

To identify whether any individual threshold was disproportionately influential, we examined each measure as a function of the shift applied to each threshold, marginalizing over all combinations of the remaining four thresholds (Fig. S11). Across all 243 configurations, the baseline-significant age effects were largely preserved: on average, 93.6% of significant findings were retained, the mean coefficient of variation in β_{age} was 0.094, and 99.8% of the β_{age} coefficient have preserved their sign. No single threshold parameter was associated with a substantial loss of significance or a large change in effect size, indicating that our findings are not unduly sensitive to the specific choice of any one boundary.

We note two limitations of this analysis. First, because the measures are marginalized over the remaining thresholds, the per-threshold summaries do not isolate potential interactions between thresholds; however, the full grid of 243 configurations does jointly span ±10% perturbations across all five thresholds simultaneously, so such interaction effects are captured in the overall distribution of outcomes. Second, the binary significance-retention measure treats all baseline-significant findings equally regardless of their proximity to the significance threshold, which is why we additionally report the continuous effect-size measure.

Supplementary Figures

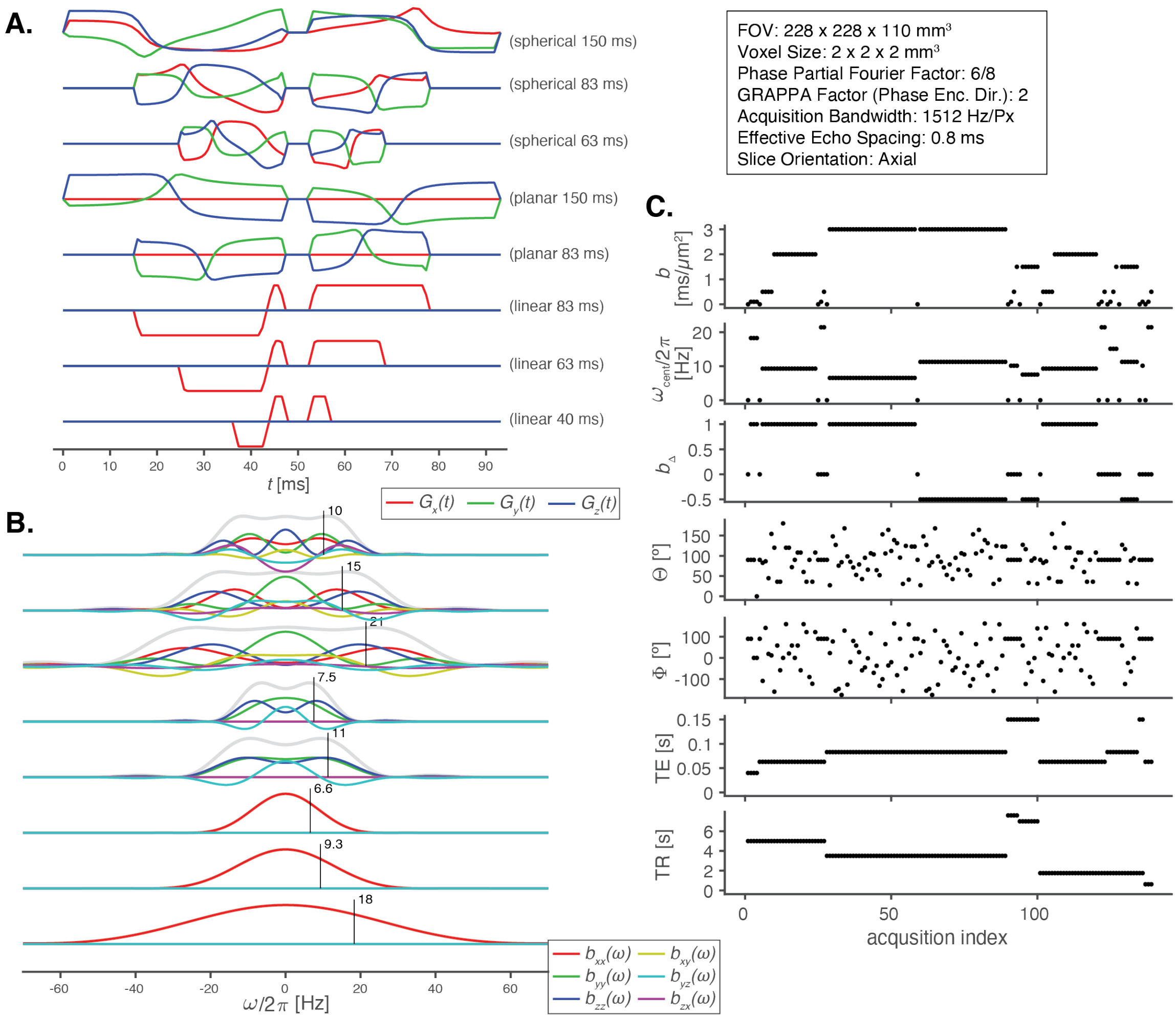

Figure S1: **MD-MRI acquisition and encoding design** (A) Time-dependent optimized diffusion gradient waveforms $G_x(t)$, $G_y(t)$, and $G_z(t)$ used to generate linear, planar, and spherical b-tensors at different echo times. (B) Corresponding diffusion spectra $b(\omega)$, illustrating the broad frequency content of the encoding waveforms. (C) Acquisition protocol showing the repetition time (TR), echo time (TE), b-tensor magnitude (b), normalized anisotropy (b_Δ; planar: -0.5, spherical: 0, linear: 1), gradient orientation angles (θ, ϕ), and centroid frequency $\omega_{\mathrm{cent}}/2\pi$, plotted as a function of acquisition index.

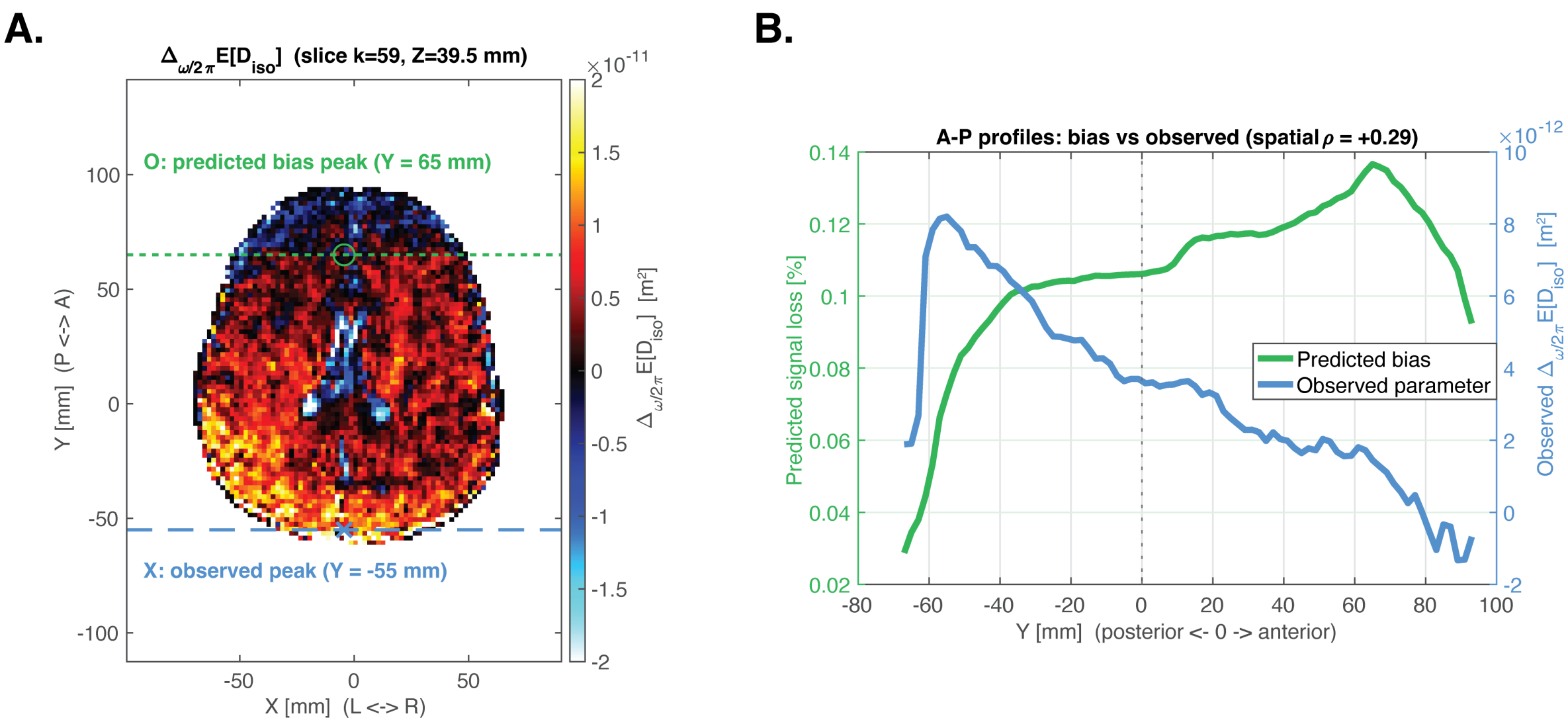

Figure S2: **Spatial comparison of predicted concomitant-field bias and observed diffusion frequency dependency.** **A.** Axial slice of the observed $\Delta_{\omega/2\pi}\mathrm{E}[D_{\mathrm{iso}}]$ map. Markers indicate the observed parameter peak ($Y = -55$ mm) and the predicted concomitant-field bias peak ($Y = +65$ mm), located on opposite anterior-posterior sides of isocenter. **B.** Anterior-posterior profiles of the observed parameter (blue, right axis) and protocol-averaged predicted bias (green, left axis), averaged over X and Z within the brain mask.

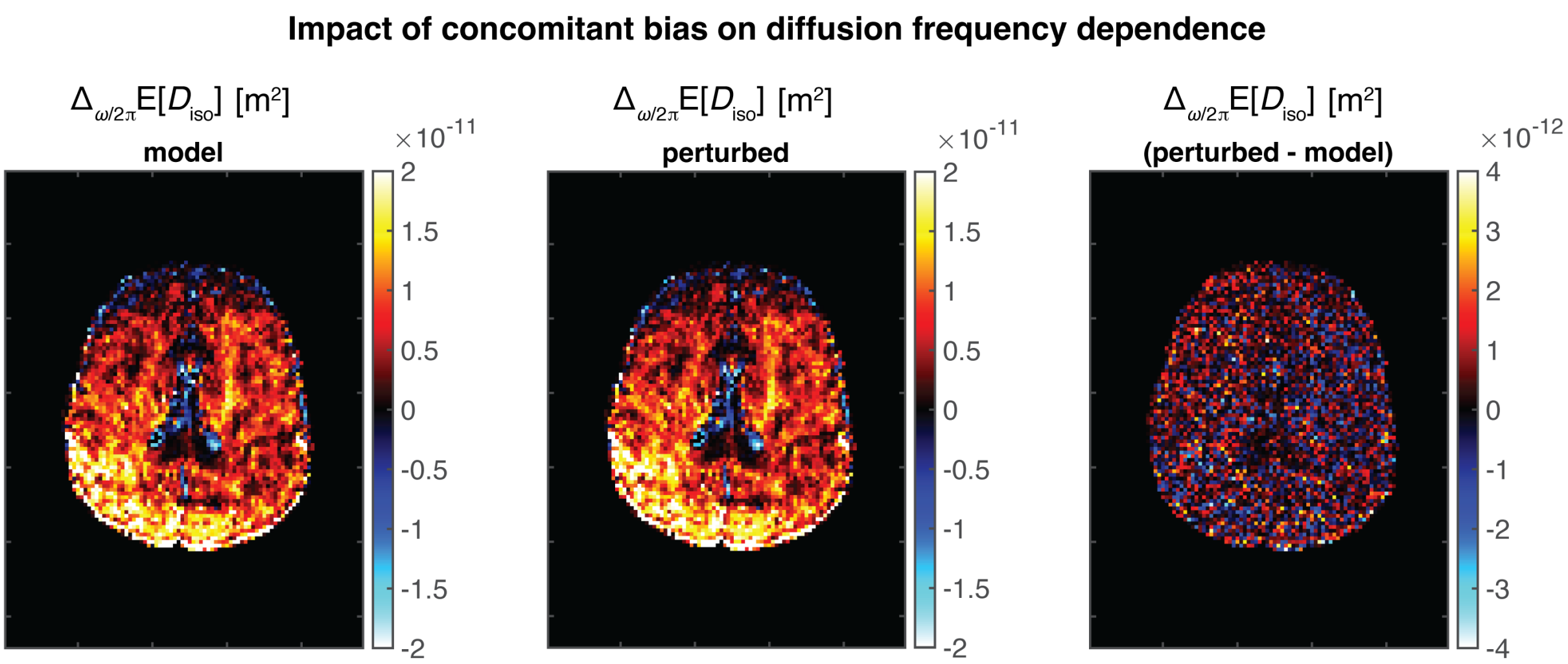

Figure S3: **Voxel-wise propagation of predicted concomitant-field bias into diffusion frequency dependency estimations.** At each brain voxel, synthetic signals for all 139 waveforms were generated from the fitted parameters, multiplied by the predicted concomitant-field attenuation, and refit. Left: unperturbed $\Delta_{\omega/2\pi}\mathrm{E}[D_{\mathrm{iso}}]$ map. Center: perturbed map. Right: voxel-wise difference (note the 10× finer color scale).

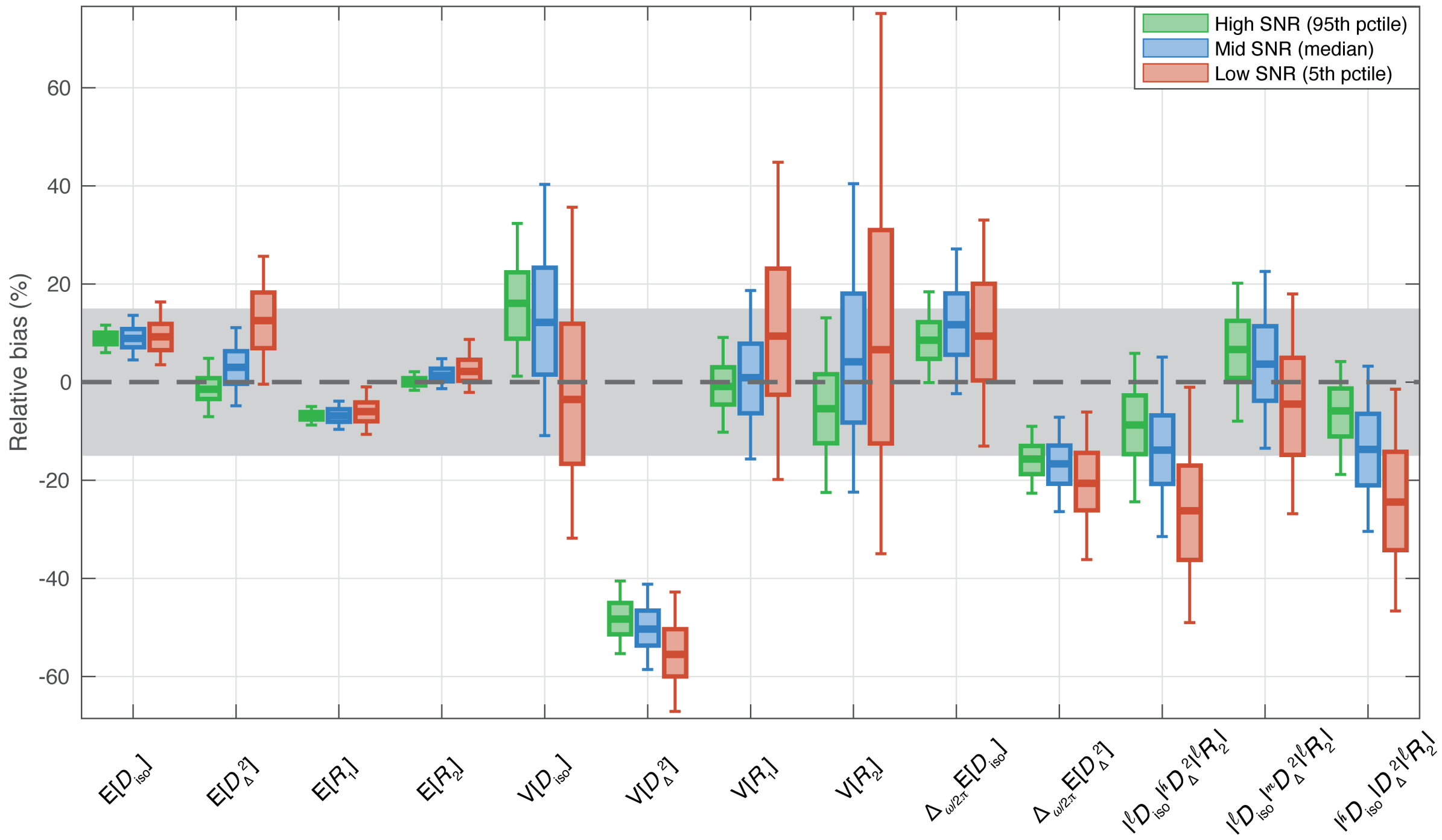

Figure S4: Grouped boxplots showing the relative bias (%) of each output parameter across 900 noise realizations at three SNR levels (green: high SNR = 542.3; blue: median SNR = 298.5; red: low SNR = 157.9). For each parameter group, boxes show the interquartile range, the horizontal line indicates the median, and whiskers extend to the 5th and 95th percentiles. The dashed horizontal line indicates zero bias. Bias below 15% (grey shaded region) was considered acceptable given the complexity of the forward model and the single canonical ground truth spectrum used. The two parameters exceeding this threshold (V[D_Δ^2], $\Delta_{\omega/2\pi}E[D_\Delta^2]$) are both related to diffusion anisotropy and their elevated bias is attributable to gradient hardware limitations of clinical MRI scanners rather than noise floor effects.

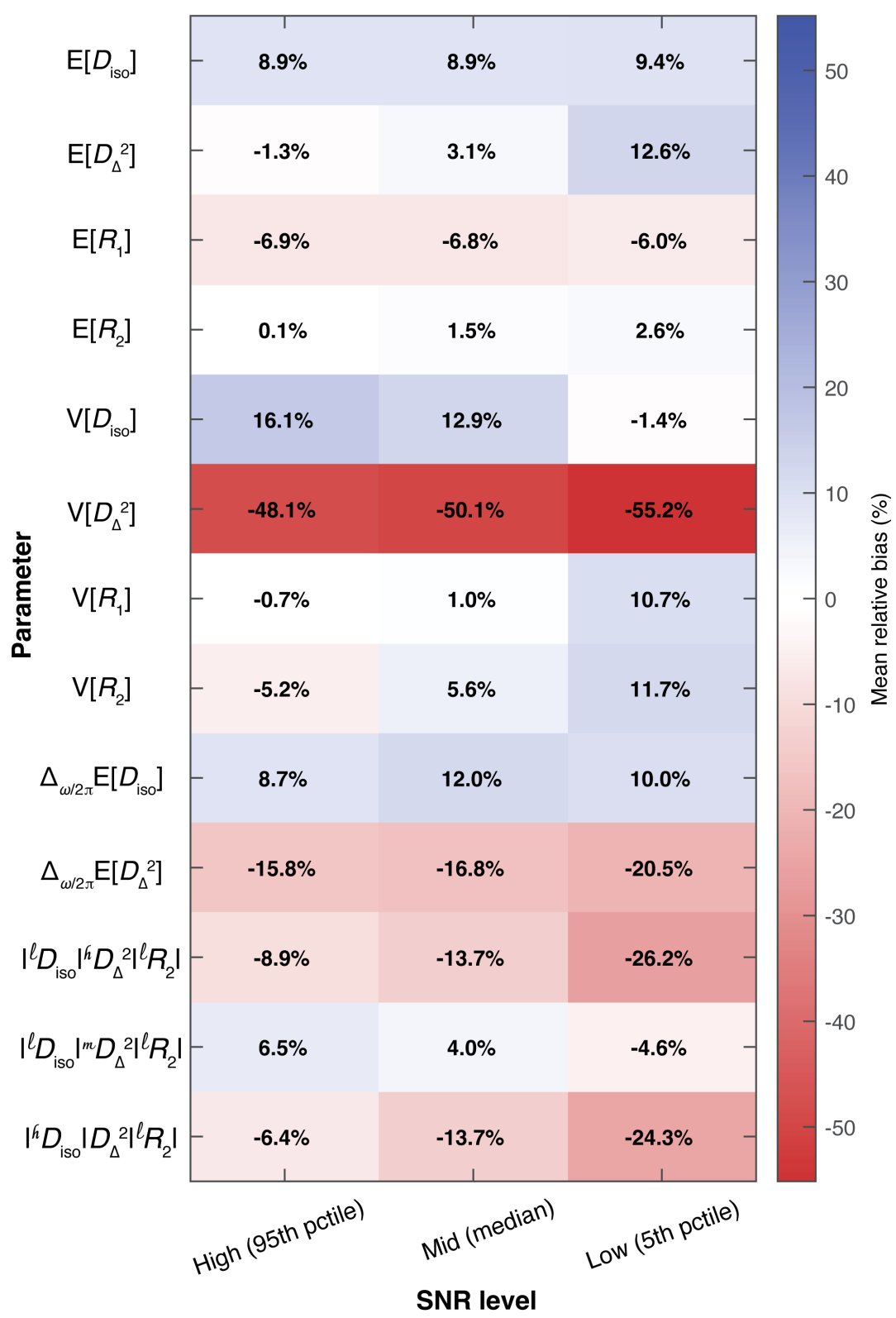

Figure S5: Each cell shows the mean relative bias (%) for one parameter (rows) at one SNR level (columns). The color scale is symmetric around zero: blue indicates positive bias (overestimation), red indicates negative bias (underestimation), and white indicates no bias. SNR levels correspond to the 5th percentile, median, and 95th percentile of the per-ROI median SNR distribution observed across subjects and ROIs (SNR = 157.9, 298.5, and 542.3 respectively). The pattern of bias is consistent across SNR levels for all parameters, confirming that estimation errors are driven by model complexity rather than by noise floor effects at the SNR levels achieved in this study.

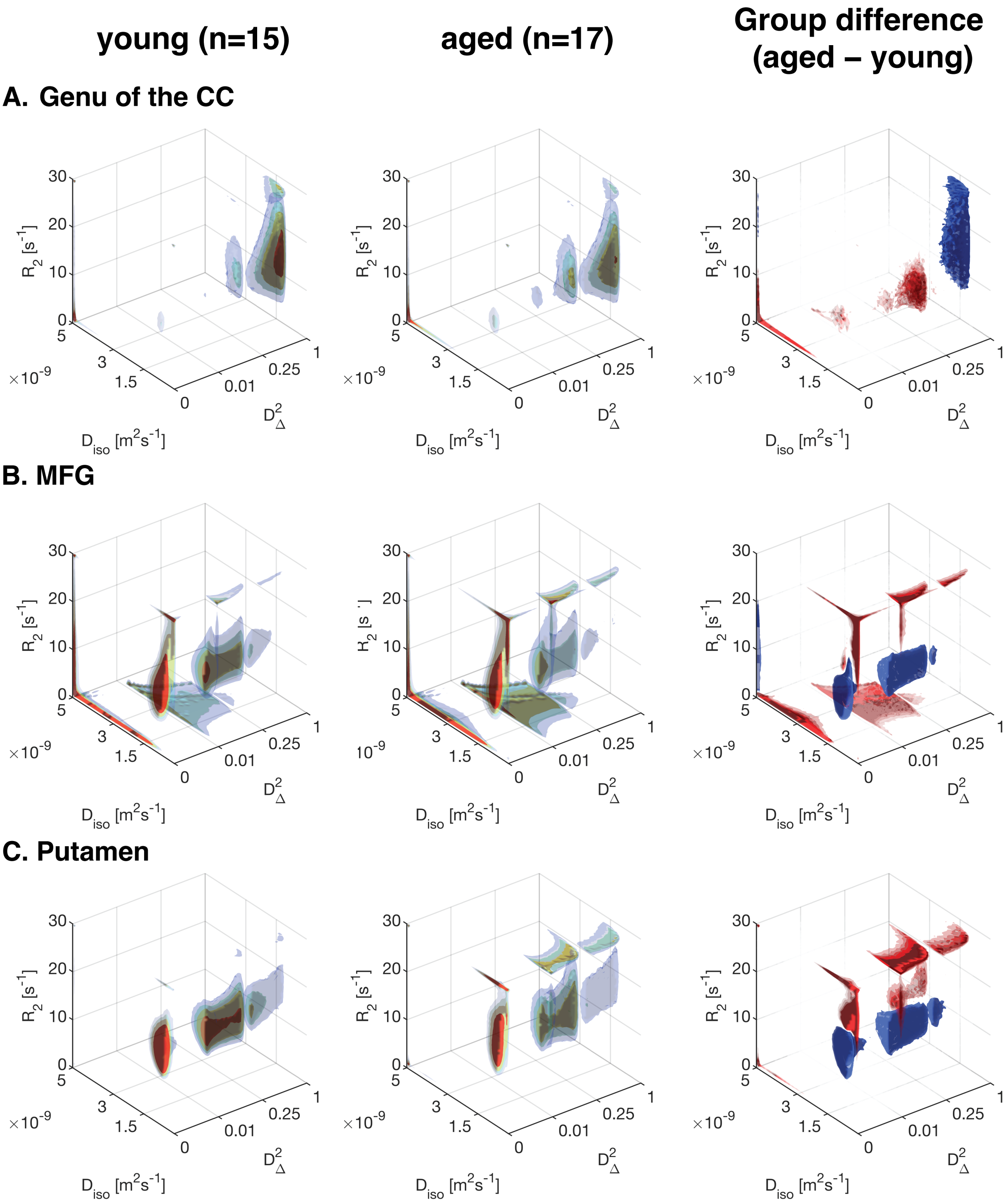

Figure S6: Age-related redistribution of joint diffusion-relaxation phenotypes across major brain tissue classes. Group-averaged three-dimensional joint D_{iso}–D^2_Δ–R_2 distributions projected at maximal frequency, ω_{min} = 21 Hz, are shown for young (left column) and aged adults (middle column) in the (A) genu of the corpus callosum, (B) middle frontal gyrus (MFG), and (C) putamen. Nested isosurfaces are shown at the 98th, 99th, 99.5th, and 99.9th percentiles of the grand-mean distribution pooled across all subjects, colored from deep blue (lowest) to red (highest) with opacity increasing cubically with percentile level. On the right column, red (positive) and blue (negative) isosurfaces show regions of difference between aged and young adults, rendered at four opacity levels spanning 50–100% of the peak absolute difference. White surface indicates zero. Across brain regions, aging is marked by multilateral microstructural shifts that reflect complex trajectories and together form a shared aging signature.

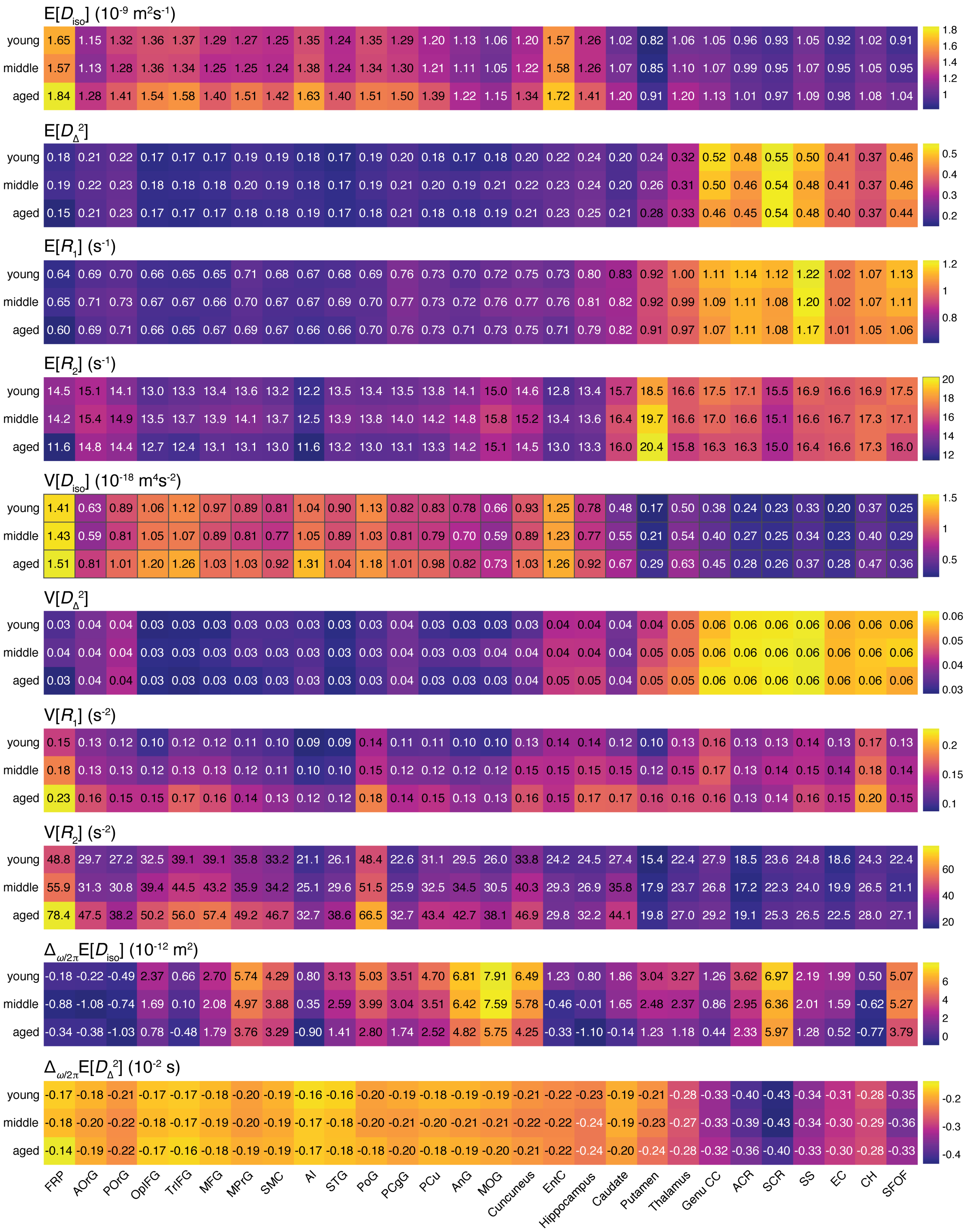

Figure S7: Region-of-interest–averaged MD-MRI diffusion–relaxation parameters, stratified by age group.

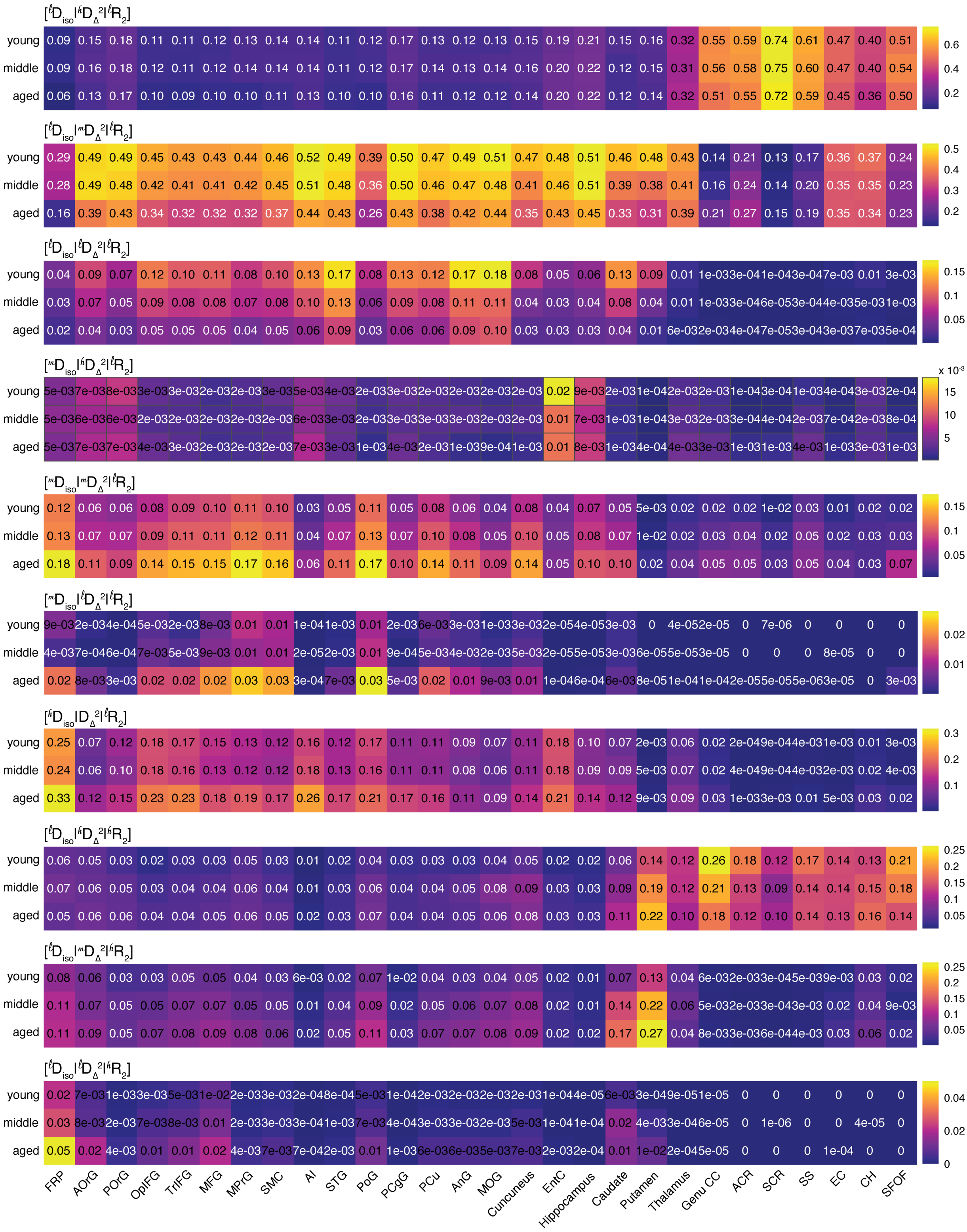

Figure S8: Region-of-interest–averaged MD-MRI microstructural phenotypes, stratified by age group. Some phenotypes have clear regional specificity; for example, both $|^{\ell}D_{\rm iso}|^{h}D_{\Delta}^{2}|^{\ell}R_{2}|$ and $|^{\ell}D_{\rm iso}|^{h}D_{\Delta}^{2}|^{h}R_{2}|$ are more prevalent in WM. Conversely, lower anisotropy subdomains, e.g., $|^{\ell}D_{\rm iso}|^{\ell}D_{\Delta}^{2}|^{\ell}R_{2}|$ or $|^{m}D_{\rm iso}|^{m}D_{\Delta}^{2}|^{\ell}R_{2}|$, mainly dominate cortical and subcortical regions.

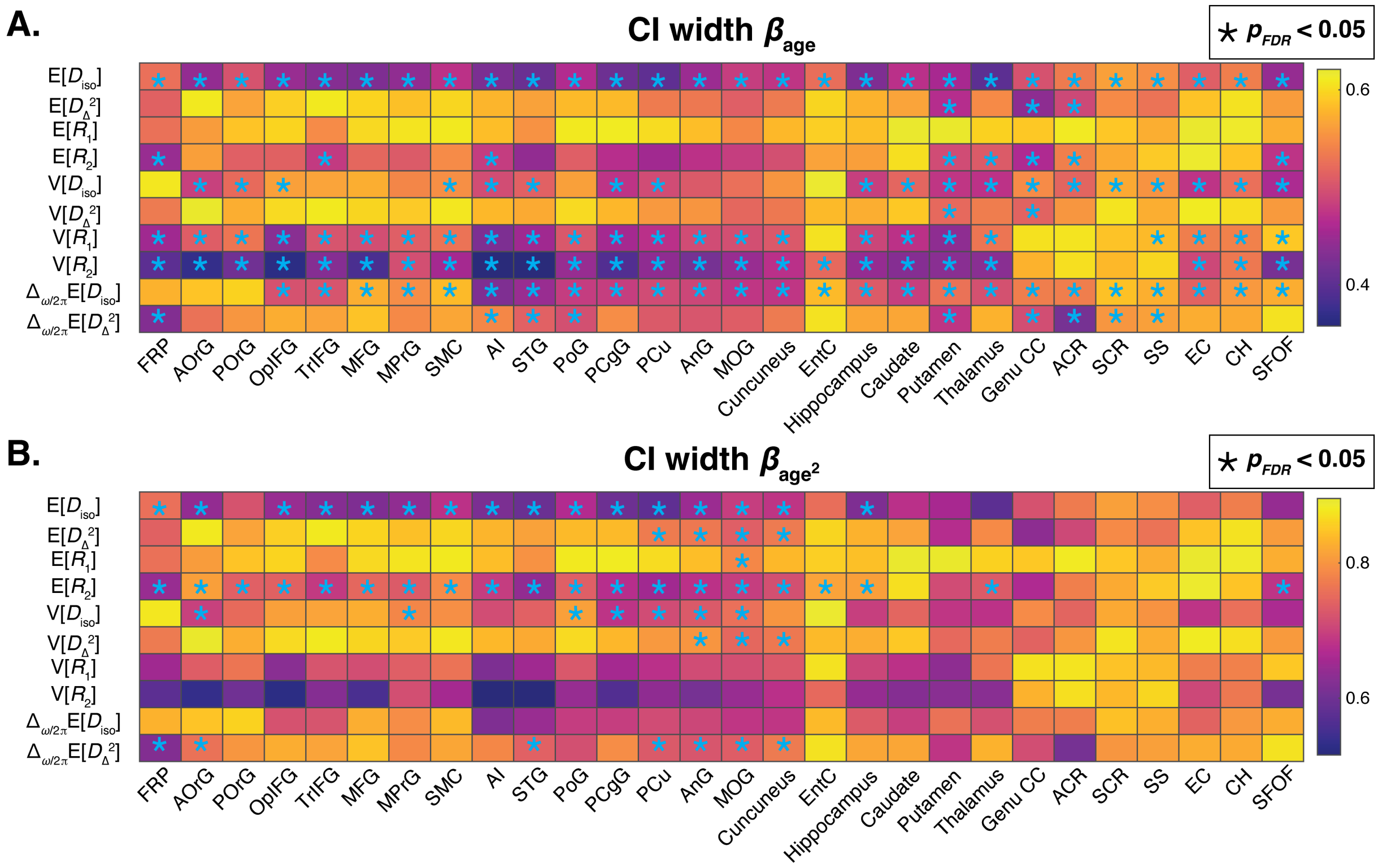

Figure S9: CI width heatmaps for the (A) linear (β_{age}) and (B) quadratic (β_{age^2}) age regression coefficients across 28 ROIs (columns) and 10 diffusion-relaxation parameters (rows). Color reflects the width of the 95% confidence interval, where lighter colors indicate greater estimation uncertainty. Asterisks denote ROI-parameter pairs reaching statistical significance after FDR correction. CI width pattern is consistent across both age terms, differing only in overall magnitude.

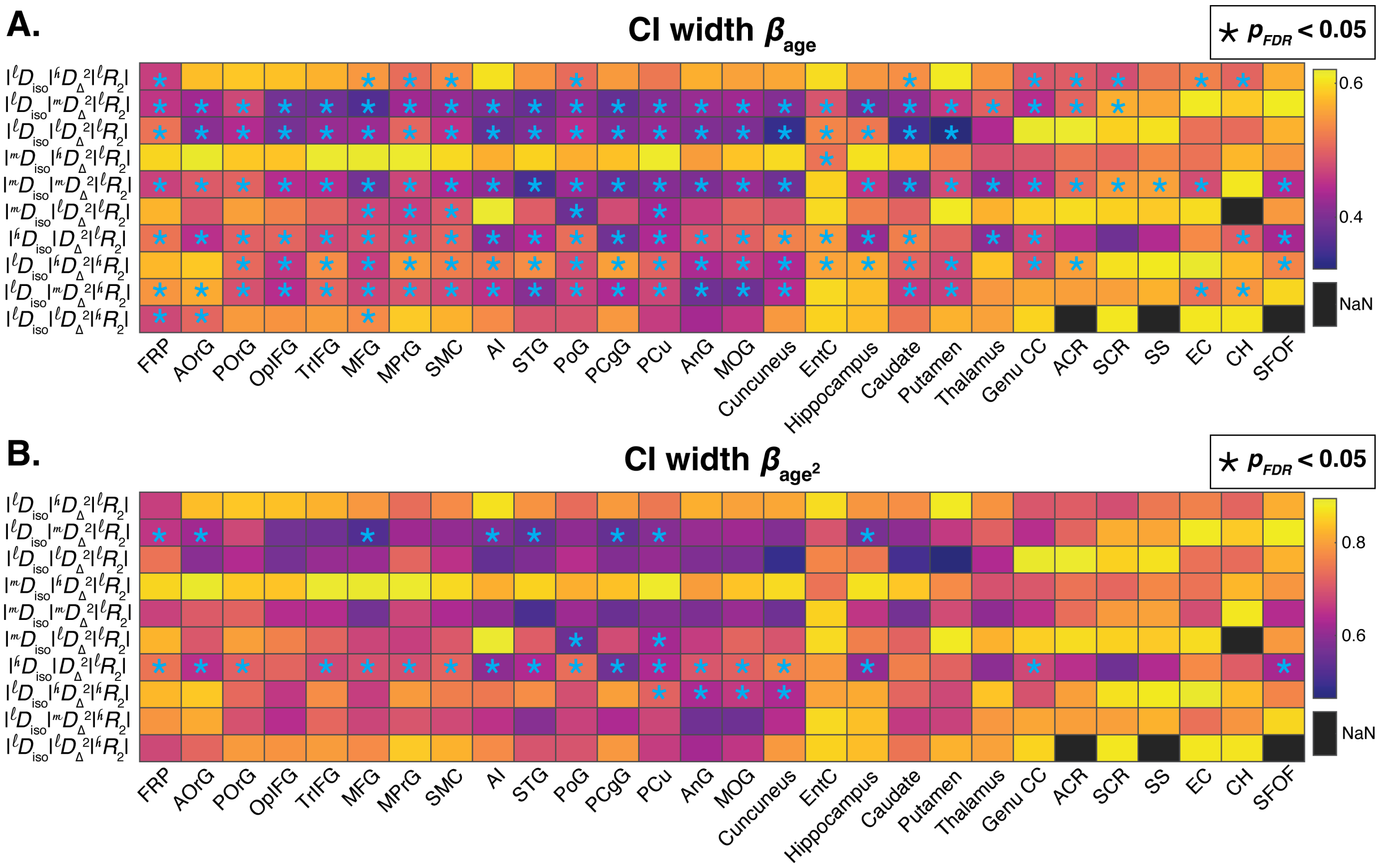

Figure S10: CI width heatmaps for the (A) linear (β_{age}) and (B) quadratic (β_{age^2}) age regression coefficients across 28 ROIs (columns) and 10 microstructural phenotypes (rows). Color reflects the width of the 95% confidence interval, where lighter colors indicate greater estimation uncertainty. Asterisks denote ROI-parameter pairs reaching statistical significance after FDR correction. CI width pattern is consistent across both age terms, differing only in overall magnitude. Black cells indicate ROI-parameter pairs with no signal variance across subjects and were excluded from analysis.

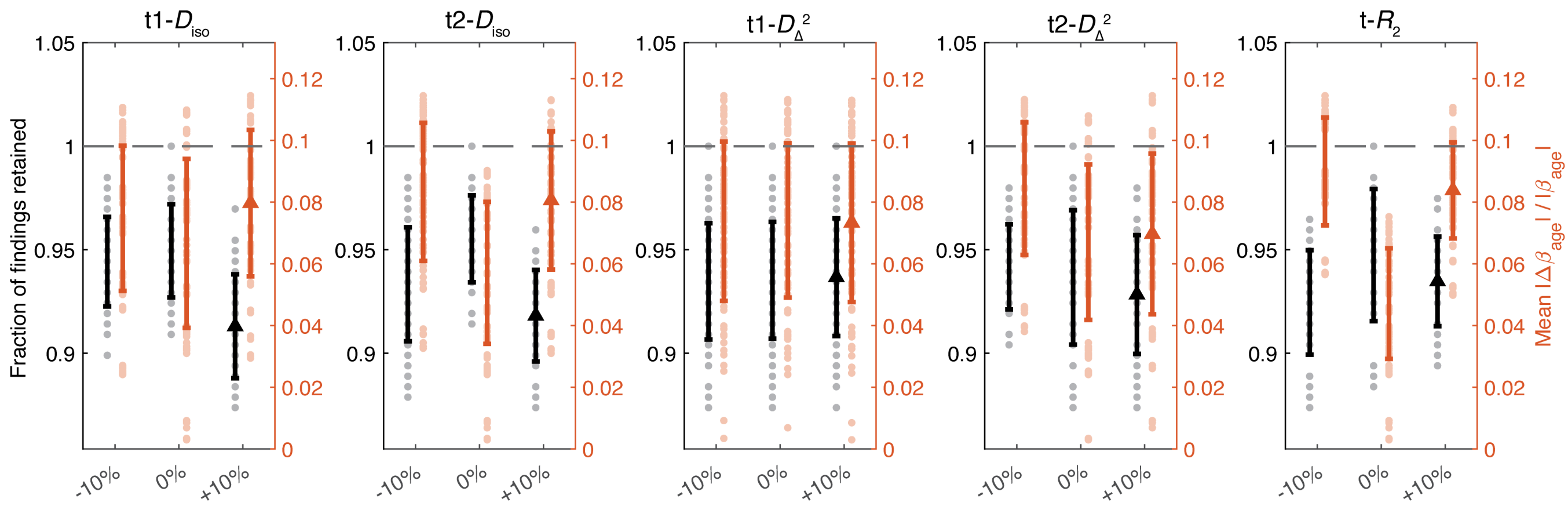

Figure S11: Robustness of age-related microstructural effects to threshold perturbation. To assess the sensitivity of our findings to the heuristic boundaries used to define the microstructural phenotypes, we systematically perturbed each of the five internal threshold parameters (two on D_{iso}: t1-D_{iso}, t2-D_{iso}; two on D_Δ^2: t1-D_Δ^2, t2-D_Δ^2; one on R_2: t-R_2) by -10%, 0%, or +10%, yielding $3^5 = 243$ unique threshold configurations. For each configuration, the full analysis pipeline was rerun across all 46 subjects, and the quadratic regression model was recomputed for every phenotype–ROI pair. Each panel shows the effect of perturbing one threshold parameter, marginalizing over all combinations of the remaining four. Left axis (black): the fraction of bin/ROI pairs that were statistically significant in the main analysis (baseline, 0% shift; $p_{FDR}<0.05$) that remained significant under perturbation. Right axis (orange): the mean relative change in the linear age coefficient, $|\Delta\beta_{age}|/|\beta_{age}|$, averaged across all baseline-significant bin/ROI pairs. Points represent individual threshold configurations at each shift level; error bars denote mean ± SD. Values near 1.0 (left) and near 0 (right) indicate that findings are robust to threshold choice.

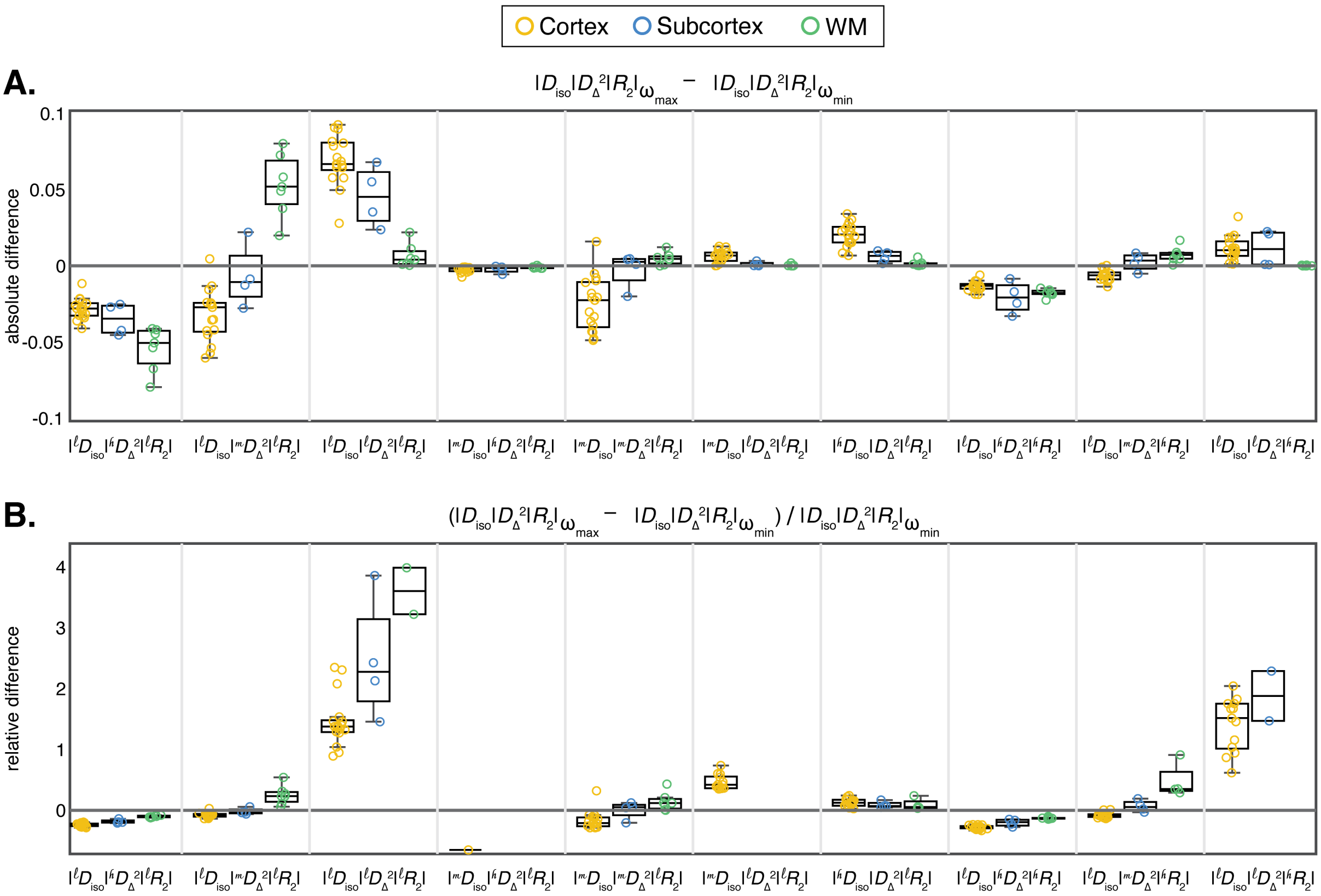

Figure S12: Diffusion frequency dependence of microstructural phenotypes. Panels A and B show the absolute and relative differences, respectively, between high- and low-frequency estimates averaged across the aged adult group. Differences are grouped and color-coded by brain region (cortical GM, subcortical GM, and WM). Phenotypes with ROI-averaged intensity under 0.01 were omitted from the plot to avoid bias.

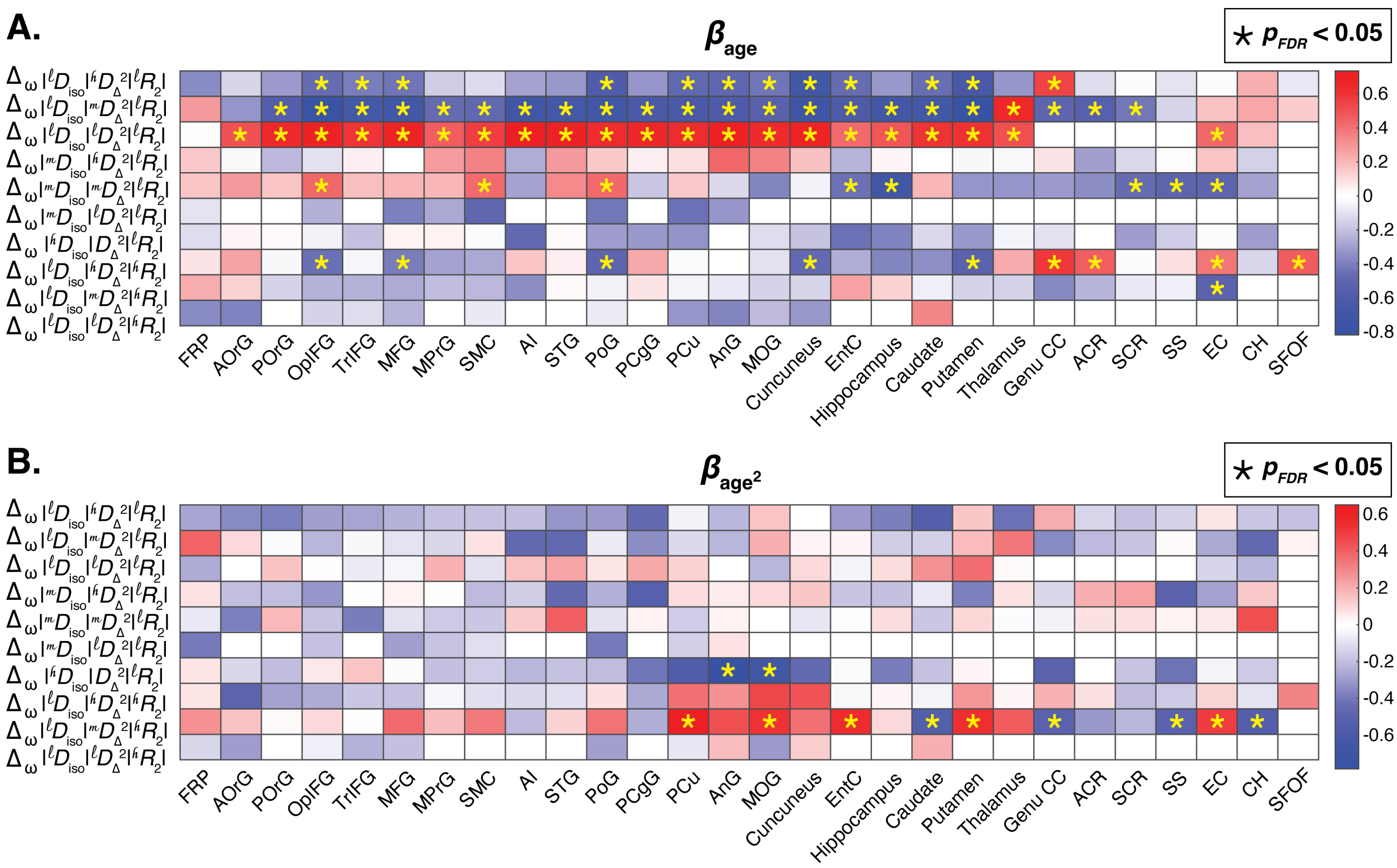

Figure S13: Linear and nonlinear age associations of frequency dependence of microstructural phenotypes across brain regions. (A) Standardized linear age coefficients (β_{age}) estimated for signal fraction frequency dependence associated with distinct diffusion-relaxation subdomains across cortical, deep GM, and WM ROIs. (B) Corresponding quadratic age coefficients (β_{age^2}) capturing nonlinear lifespan trajectories. Phenotypes with ROI-averaged intensity under 0.01 were omitted from the plot to avoid bias.

References

[1] Jan Martin, Alexis Reymbaut, Manuel Schmidt, Arnd Doerfler, Michael Uder, Frederik Bernd Laun, and Daniel Topgaard. Nonparametric d-r1-r2 distribution mri of the living human brain. *NeuroImage*, 245:118753, 12 2021. ISSN 10538119. doi: 10.1016/j.neuroimage.2021.118753.

[2] Andreas Wetscherek, Bram Stieltjes, and Frederik Bernd Laun. Flow-compensated intravoxel incoherent motion diffusion imaging. *Magnetic Resonance in Medicine*, 74:410–419, 8 2015. ISSN 07403194. doi: 10.1002/mrm.25410.

[3] Jens Sjölund, Filip Szczepankiewicz, Markus Nilsson, Daniel Topgaard, Carl-Fredrik Westin, and Hans Knutsson. Constrained optimization of gradient waveforms for generalized diffusion encoding. *Journal of Magnetic Resonance*, 261: 157–168, 12 2015. ISSN 10907807. doi: 10.1016/j.jmr.2015.10.012.

[4] Eric Seth Michael, Franciszek Hennel, and Klaas Paul Pruessmann. Evaluating diffusion dispersion across an extended range of b-values and frequencies: Exploiting gap-filled OGSE shapes, strong gradients, and spiral readouts. *Magnetic Resonance in Medicine*, 87(6):2710–2723, June 2022. ISSN 0740-3194, 1522-2594. doi: 10.1002/mrm.29161.

[5] Filip Szczepankiewicz, Carl-Fredrik Westin, and Markus Nilsson. Gradient waveform design for tensor-valued encoding in diffusion MRI. *Journal of Neuroscience Methods*, 348:109007, January 2021. ISSN 01650270. doi: 10.1016/j.jneumeth. 2020.109007.

[6] Jessica T. E. Johnson, M. Okan Irfanoglu, Eppu Manninen, Thomas J. Ross, Yihong Yang, Frederik B. Laun, Jan Martin, Daniel Topgaard, and Dan Benjamini. In vivo disentanglement of diffusion frequency-dependence, tensor shape, and relaxation using multidimensional MRI. *Human Brain Mapping*, 45(7):e26697, May 2024. ISSN 1065-9471, 1097-0193. doi: 10.1002/hbm.26697.

[7] Jelle Veraart, Dmitry S Novikov, Daan Christiaens, Benjamin Ades-Aron, Jan Sijbers, and Els Fieremans. Denoising of diffusion mri using random matrix theory. *Neuroimage*, 142:394–406, 2016.

[8] Elias Kellner, Bibek Dhital, Valerij G Kiselev, and Marco Reisert. Gibbs-ringing artifact removal based on local subvoxel-shifts. *Magnetic resonance in medicine*, 76(5):1574–1581, 2016.

[9] Hong-Hsi Lee, Dmitry S Novikov, and Els Fieremans. Removal of partial fourier-induced gibbs (rpg) ringing artifacts in mri. *Magnetic resonance in medicine*, 86(5):2733–2750, 2021.

[10] Carlo Pierpaoli, Lindsay Walker, Mustafa Okan Irfanoglu, Alan Barnett, Peter Basser, Lin-Ching Chang, C Koay, Sinisia Pajevic, Gustavo Rohde, Joelle Sarlls, et al. Tortoise: an integrated software package for processing of diffusion mri data. In *ISMRM 18th annual meeting*, volume 1597. ISMRM Germany, 2010.

[11] M Okan Irfanoglu, Pooja Modi, Amritha Nayak, Elizabeth B Hutchinson, Joelle Sarlls, and Carlo Pierpaoli. Dr-buddi (diffeomorphic registration for blip-up blip-down diffusion imaging) method for correcting echo planar imaging distortions. *Neuroimage*, 106:284–299, 2015.

[12] Kurt G Schilling, Justin Blaber, Yuankai Huo, Allen Newton, Colin Hansen, Vishwesh Nath, Andrea T Shafer, Owen Williams, Susan M Resnick, Baxter Rogers, et al. Synthesized b0 for diffusion distortion correction (synb0-disco). *Magnetic resonance imaging*, 64:62–70, 2019.

[13] Markus Nilsson, Filip Szczepankiewicz, Björn Lampinen, André Ahlgren, Jo ao P. de Almeida Martins, Samo Lasic, Carl-Fredrik Westin, and Daniel Topgaard. An open-source framework for analysis of multidimensional diffusion mri data implemented in matlab. Proceedings of the International Society for Magnetic Resonance in Medicine, Paris, France, 2018.

[14] Dan Benjamini and Peter J. Basser. Magnetic resonance microdynamic imaging reveals distinct tissue microenvironments. *NeuroImage*, 163:183–196, 12 2017. ISSN 10538119. doi: 10.1016/j.neuroimage.2017.09.033.

[15] Dan Benjamini and Peter J. Basser. Multidimensional correlation mri. *NMR in Biomedicine*, 33, 12 2020. ISSN 0952-3480. doi: 10.1002/nbm.4226.

[16] Omar Narvaez, Leo Svenningsson, Maxime Yon, Alejandra Sierra, and Daniel Topgaard. Massively multidimensional diffusion-relaxation correlation mri. *Frontiers in Physics*, 9:793966, 2022.

[17] Petrik Galvosas and Paul T Callaghan. Multi-dimensional inverse laplace spectroscopy in the nmr of porous media. *Comptes Rendus Physique*, 11(2):172–180, 2010.

[18] Diana Bernin and Daniel Topgaard. Nmr diffusion and relaxation correlation methods: New insights in heterogeneous materials. *Current opinion in colloid & interface science*, 18(3):166–172, 2013.

[19] João P de Almeida Martins and Daniel Topgaard. Multidimensional correlation of nuclear relaxation rates and diffusion tensors for model-free investigations of heterogeneous anisotropic porous materials. *Scientific reports*, 8(1):2488, 2018.

[20] Matteo Bastiani, Michiel Cottaar, Sean P. Fitzgibbon, Sana Suri, Fidel Alfaro-Almagro, Stamatios N. Sotiropoulos, Saad Jbabdi, and Jesper L.R. Andersson. Automated quality control for within and between studies diffusion MRI data using a non-parametric framework for movement and distortion correction. *NeuroImage*, 184:801–812, January 2019. ISSN 10538119. doi: 10.1016/j.neuroimage.2018.09.073.

[21] Kurt G. Schilling, Karthik Ramadass, Viljami Sairanen, Michael E. Kim, Francois Rheault, Nancy Newlin, Tin Nguyen, Laura Barquero, Micah D'archangel, Chenyu Gao, Ema Topolnjak, Nazirah Mohd Khairi, Derek Archer, Lori L. Beason-Held, Susan M. Resnick, Timothy Hohman, Laurie Cutting, Julie Schneider, Lisa L. Barnes, David A. Bennett, Konstantinos Arfanakis, Sophia Vinci-Booher, Marilyn Albert, The BIOCARD Study Team, The Alzheimer's Disease Neuroimaging Initiative (ADNI), Aging Brain: Vasculature, Ischemia, and Behavior (ABVIB), Daniel Moyer, and Bennett A. Landman. Head Motion in Diffusion Magnetic Resonance Imaging: Quantification, Mitigation, and Structural Associations in Large, Cross-Sectional Datasets Across the Lifespan. *Human Brain Mapping*, 46(3):e70143, February 2025. ISSN 1065-9471, 1097-0193. doi: 10.1002/hbm.70143.

[22] HáKon Gudbjartsson and Samuel Patz. The rician distribution of noisy mri data. *Magnetic Resonance in Medicine*, 34(6): 910–914, December 1995. ISSN 0740-3194, 1522-2594. doi: 10.1002/mrm.1910340618.

[23] Jelle Veraart, Jan Sijbers, Stefan Sunaert, Alexander Leemans, and Ben Jeurissen. Weighted linear least squares estimation of diffusion MRI parameters: Strengths, limitations, and pitfalls. *NeuroImage*, 81:335–346, November 2013. ISSN 10538119. doi: 10.1016/j.neuroimage.2013.05.028.

[24] C. A. Baron, R. M. Lebel, A. H. Wilman, and C. Beaulieu. The effect of concomitant gradient fields on diffusion tensor imaging. *Magnetic Resonance in Medicine*, 68(4):1190–1201, October 2012. ISSN 0740-3194, 1522-2594. doi: 10.1002/mrm.24120.

[25] Filip Szczepankiewicz, Carl-Fredrik Westin, and Markus Nilsson. Maxwell-compensated design of asymmetric gradient waveforms for tensor-valued diffusion encoding. *Magnetic Resonance in Medicine*, 82(4):1424–1437, October 2019. ISSN 0740-3194, 1522-2594. doi: 10.1002/mrm.27828.

[26] Alexis Reymbaut, Paolo Mezzani, João P. De Almeida Martins, and Daniel Topgaard. Accuracy and precision of statistical descriptors obtained from multidimensional diffusion signal inversion algorithms. *NMR in Biomedicine*, 33(12):e4267, December 2020. ISSN 0952-3480, 1099-1492. doi: 10.1002/nbm.4267.

[27] Joon Sik Park, Eppu Manninen, Yihong Yang, and Dan Benjamini. Informed Dictionary-Guided Monte Carlo Inversion for Robust and Reproducible Multidimensional MRI. *Magnetic Resonance in Medicine*, page mrm.70228, December 2025. ISSN 0740-3194, 1522-2594. doi: 10.1002/mrm.70228.
